# Highly crystalline and water-wettable benzobisthiazole-based covalent organic frameworks for enhanced photocatalytic hydrogen production

**DOI:** 10.1093/nsr/nwac171

**Published:** 2022-08-18

**Authors:** Wei Huang, Yongpan Hu, Zhengyuan Qin, Yujin Ji, Xuan Zhao, Yunling Wu, Qing He, Youyong Li, Chunfeng Zhang, Jun Lu, Yanguang Li

**Affiliations:** Institute of Functional Nano and Soft Materials (FUNSOM), Soochow University, Suzhou 215123, China; Jiangsu Key Laboratory for Advanced Negative Carbon Technologies, Soochow University, Suzhou 215123, China; Institute of Functional Nano and Soft Materials (FUNSOM), Soochow University, Suzhou 215123, China; Jiangsu Key Laboratory for Advanced Negative Carbon Technologies, Soochow University, Suzhou 215123, China; National Laboratory of Solid State Microstructures, School of Physics, and Collaborative Innovation Center for Advanced Microstructures, Nanjing University, Nanjing 210093, China; Institute of Functional Nano and Soft Materials (FUNSOM), Soochow University, Suzhou 215123, China; Jiangsu Key Laboratory for Advanced Negative Carbon Technologies, Soochow University, Suzhou 215123, China; Institute of Functional Nano and Soft Materials (FUNSOM), Soochow University, Suzhou 215123, China; Jiangsu Key Laboratory for Advanced Negative Carbon Technologies, Soochow University, Suzhou 215123, China; Institute of Functional Nano and Soft Materials (FUNSOM), Soochow University, Suzhou 215123, China; Jiangsu Key Laboratory for Advanced Negative Carbon Technologies, Soochow University, Suzhou 215123, China; Institute of Functional Nano and Soft Materials (FUNSOM), Soochow University, Suzhou 215123, China; Jiangsu Key Laboratory for Advanced Negative Carbon Technologies, Soochow University, Suzhou 215123, China; Institute of Functional Nano and Soft Materials (FUNSOM), Soochow University, Suzhou 215123, China; Jiangsu Key Laboratory for Advanced Negative Carbon Technologies, Soochow University, Suzhou 215123, China; Macao Institute of Materials Science and Engineering (MIMSE), MUST-SUDA Joint Research Center for Advanced Functional Materials, Macau University of Science and Technology, Macau, China; National Laboratory of Solid State Microstructures, School of Physics, and Collaborative Innovation Center for Advanced Microstructures, Nanjing University, Nanjing 210093, China; College of Chemical and Biological Engineering, Zhejiang University, Hangzhou 310027, China; Institute of Functional Nano and Soft Materials (FUNSOM), Soochow University, Suzhou 215123, China; Jiangsu Key Laboratory for Advanced Negative Carbon Technologies, Soochow University, Suzhou 215123, China; Macao Institute of Materials Science and Engineering (MIMSE), MUST-SUDA Joint Research Center for Advanced Functional Materials, Macau University of Science and Technology, Macau, China

**Keywords:** covalent organic frameworks, photocatalysis, hydrogen evolution, benzobisthiazole, water-wettable

## Abstract

Two-dimensional covalent organic frameworks are promising for photocatalysis by virtue of their structural and functional diversity, but generally suffer from low activities relative to their inorganic competitors. To fulfill their full potential requires a rational tailoring of their structures at different scales as well as their surface properties. Herein, we demonstrate benzobisthiazole-based covalent organic frameworks as a superior photocatalyst for hydrogen production. The product features high crystallinity with ordered 2.5-nm-wide cylindrical mesopores and great water wettability. These structural advantages afford our polymeric photocatalyst with fast charge carrier dynamics as evidenced by a range of spectroscopic characterizations and excellent catalytic performances when suspended in solution or supported on melamine foams. Under visible-light irradiation, it enables efficient and stable hydrogen evolution with a production rate of 487 μmol h^−1^ (or a mass-specific rate of 48.7 mmol g^−1^ h^−1^)—far superior to the previous state of the art. We also demonstrate that hydrogen production can be stoichiometrically coupled with the oxidation conversion of biomass as exemplified by the conversion of furfuryl alcohol to 2-furaldehyde.

## Teaser

Semiconducting benzobisthiazole-based covalent organic frameworks were prepared and reported. Its high crystallinity, great water-wettability and fast charge separation dynamics cooperatively endow the material with an extraordinary H2 evolution rate of 48.7 mmol g1 h1 under visible light irradiation.

## INTRODUCTION

The conversion of abundant solar energy into clean hydrogen fuels via photocatalytic water splitting is a promising pathway to accelerate our transition toward a sustainable energy future [1]. The key lies in the rational design of semiconductor photocatalysts ideally with broad light-absorption ranges, rapid charge separation and large accessible surface areas. Since the first report in 1972 [2], research attention has been predominantly focused on inorganic semiconductors such as TiO_2_, CdS and so on [3–6]. Polymeric photocatalysts, on the other hand, are unique for their Earth-abundant compositions and structural diversity, and therefore have tremendous potential to rival or even outperform their inorganic competitors [7–11]. By properly choosing the building blocks and building sequences, their optoelectronic properties might be rationally tailored to demand. However, polymeric photocatalysts are far less explored.

Among a number of emerging polymeric candidates, ordered 2D covalent organic frameworks (2D-COFs) attract particular attention [12–17]. They are formed by the ordered stacking of molecular layers and usually possess periodic columnar π-arrays that can facilitate interlayer charge separation and transfer. Despite much exciting progress in this direction since the pioneering work by Lotsch and co-workers in 2014 [18], attainable photocatalytic performances of 2D-COFs remain very limited (mostly <10 mmol g^−1^ h^−1^) [13]. This is mainly due to the large binding energy of their Frenkel excitons and consequent problematic exciton dissociation [8,19]; the hydrophobic nature of their π-conjugated aromatic backbones often causes their large inner porosity to be inaccessible to water [20,21]. In order to tackle the above challenges, synthetic and structural modifications are desired to promote the structural crystallinity and polarity of 2D-COFs.

Incorporating heteroatom-rich moieties into the organic frameworks of 2D-COFs can effectively increase their water wettability, expose abundant inner surface sites and facilitate exciton dissociation [8,22,23]. Here, we demonstrate the critical roles of benzobisthiazole (BBT) units in improving the photocatalytic properties of 2D-COFs: it is a nitrogen- and sulfur-containing aromatic heterocycle that may increase the product surface wettability; its rigid and planar configuration would promote structural crystallinity and enhance the intralayer electron delocalization and interlayer π–π stacking. Thanks to its high crystallinity and great water wettability, the resulting BBT-containing 2D-COF exhibits outstanding photocatalytic performance.

## RESULTS AND DISCUSSION

### Synthesis and characterizations of benzobisthiazole-based 2D-COF

**Figure 1.**
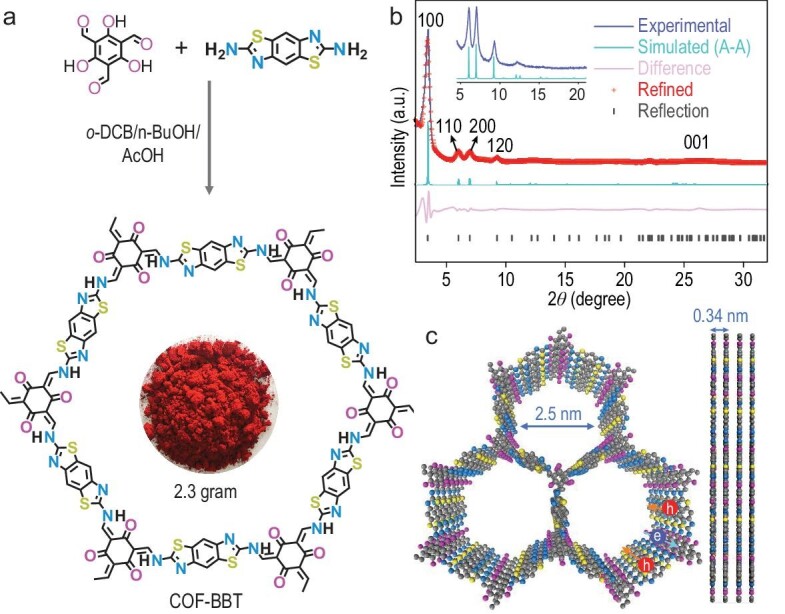
Synthesis and molecular topology of COF-BBT. (a) Schematic synthetic procedure toward COF-BBT; the inset illustrates the powdery product from a single reaction batch; (b) experimental and simulated XRD patterns of COF-BBT together with its Rietveld refinement result; (c) schematic structure of COF-BBT viewed from the top and the side.

Figure 1a schematically illustrates the synthesis of the benzobisthiazole-based 2D-COF (COF-BBT) from the condensation between 2.6-diaminebenzobisthiazole (DABBT) and 1,3,5-triformylphloroglucinol (TFP) in the presence of acetic acid as the catalyst. In Schiff-base reactions, the acid catalyst is known to favor the reversible cross-linking of individual building blocks that provides a self-correction mechanism to gradually heal defects and form ordered structures over time [24]. Here, the low oxygen reactivity of primary amines in DABBT (owing to the electron-deficient nature of benzobisthiazole) allows the reaction to proceed without the inert gas protection and renders our synthetic procedure significantly more straightforward and scalable compared to the syntheses of other similar COF materials [25]. For control experiments, we also prepared an amorphous benzobisthiazole-based covalent organic polymer (COP-BBT) in the absence of acetic acid, as well as a model molecule from the reaction of TFP and 2-aminobenzothiazole.

The cross-linked molecular structures of COF-BBT and COP-BBT were first verified using Fourier-transform infrared (FTIR) spectroscopy, nuclear magnetic resonance (NMR) spectroscopy and X-ray photoelectron spectroscopy (XPS). As shown in Supplementary Fig. S1, the absence of the stretching vibrations of aldehyde (–CH=O) at 1636 cm^−1^ or amine (–NH_2_) at 3392–3276 cm^−1^ in the FTIR spectra of COF-BBT and COP-BBT suggests the complete condensation of the starting monomers. Two new peaks instead emerge at 1602 and 1565 cm^−1^, corresponding to the ketone (C=O) and C=C bonding, respectively, of the β-ketoenamine linkage. Their solid-state ^13^C NMR spectra further reveal the presence of the ketone carbon at 184 ppm (Supplementary Fig. S2). Moreover, the N 1s XPS spectrum of COF-BBT exhibits two intensive peaks at 399.0 and 400.6 eV that can be ascribed to the C–NH bond in the enamine and the C=N bond in the BBT units, respectively (Supplementary Fig. S3). Their spectral fingerprints are also identical to those of the model molecule. All these results unambiguously indicate the formation of β-ketoenamine linkages and the incorporation of BBT units into the organic skeletons. The X-ray diffraction (XRD) pattern of COF-BBT exhibits intense diffraction peaks that can be perfectly simulated by the eclipsed (A–A) stacking of molecular layers (Fig. 1b and Supplementary Fig. S4). The strongest peak at 2θ = 3.5° is assignable to the (100) diffraction and evidences the long-range structural ordering with a periodic pore-to-pore distance of 25.2 Å. The interlayer distance of COF-BBT is estimated to be 3.4 Å from its (001) diffraction at 2θ = 26.3^o^. Compared to the staggered (A–B) configuration, such a π–π cofacial assembly would facilitate the interlayer charge separation and transfer during photocatalysis (Fig. 1c) [26]. By contrast, the XRD of COP-BBT is largely featureless and thereby attests to its amorphous nature despite the same building units as COF-BBT (Supplementary Fig. S5).

**Figure 2.**
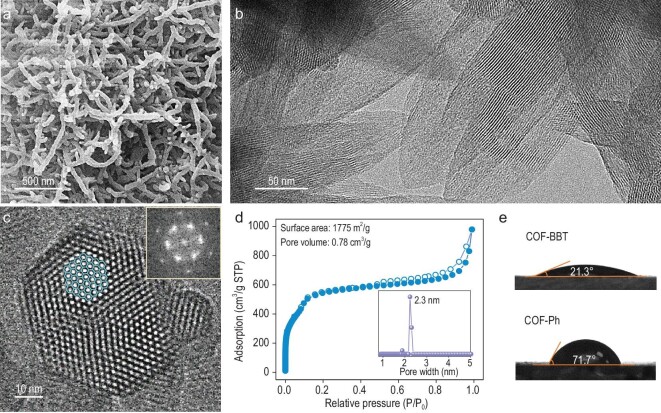
Nanostructure and surface property of COF-BBT. (a) SEM image and (b and c) TEM images of COF-BBT nanofibers; the inset of (c) is the fast Fourier-transform (FTT) pattern of the hexagonal mesopore arrangement at the nanofiber cross section; (d) N_2_ isotherm of COF-BBT; the inset shows the corresponding pore size distribution curve; (e) water contact angles of COF-BBT and COF-Ph.

Scanning electron microscopy (SEM) imaging of COF-BBT shows that it consists of nanofibers of ∼100 nm in diameter and >1 μm in length (Fig. 2a). Transmission electron microscopy (TEM) analysis corroborates the high structural crystallinity. Its 1D mesoporous channels are observed to extend in parallel along the nanofiber long-axis, which indicates that individual nanofibers are formed from the stacking layers along the [001] direction (Fig. 2b). The TEM image of a nanofiber cross section clearly unveils the hexagonal arrangement of these 1D channels (Fig. 2c). Their width is measured to be ∼2.5 nm, in good agreement with above XRD analysis. N_2_ sorption measurement shows that COF-BBT has a large surface area of 1775 m^2^ g^−1^ and a total pore volume of 0.78 cm^3^ g^−1^ (Fig. 2d). Its corresponding pore size distribution curve exhibits a sharp peak at 2.3 nm (Fig. 2d, inset). By contrast, COP-BBT shows a similar fibrous morphology but lacks long-range structural ordering under TEM (Supplementary Fig. S6). It has a considerably smaller surface area of 420 m^2^ g^−1^ and broad pore size distribution owing to its disordered molecular frameworks (Supplementary Fig. S7).

Further, it is worth highlighting that our samples have excellent chemical and thermal stability (Supplementary Figs S8 and S9) and great surface wettability essential to aqueous photocatalysis. Typical polymeric photocatalysts have water contact angles in the range of 60–110^o^ [20]. Here, thin films of our COF-BBT and COP-BBT are measured to have a small water contact angle of 21^o^ and 28^o^, respectively (Fig. 2e and Supplementary Figs S10 and S11). This is due to the incorporation of heteroatom-rich BBT units, giving rise to the increased structural polarity and water affinity. As side evidence, we also prepared a 2D-COF (COF-Ph) containing benzene units instead of BBT following a previous publication [27]. Its structural characterizations are summarized in Supplementary Fig. S12. COF-Ph thin film exhibits a relatively large water contact angle of 72^o^. Moreover, water sorption isotherms also corroborate the much-improved surface wettability of COF-BBT (694 cm^3^ g^−1^ or 56 wt% at standard temperature and pressure (STP)) and COP-BBT (452 cm^3^ g^−1^ or 36 wt% at STP) compared to COF-Ph (183 cm^3^ g^−1^ or 15 wt% at STP) (Supplementary Fig. S13).

**Figure 3.**
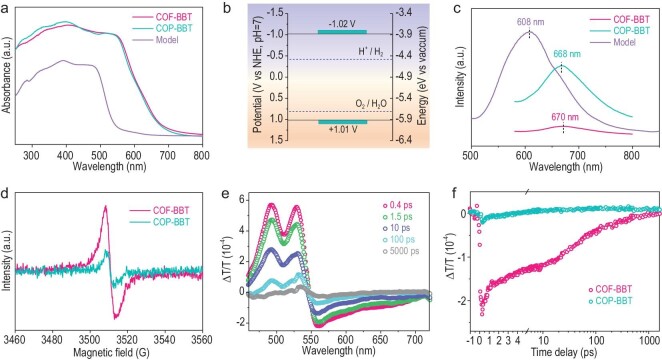
Optical properties and charge dynamics analysis. (a) UV–Vis diffuse reflectance spectra of COF-BBT, COP-BBT and the model molecule; (b) HOMO and LUMO positions of COF-BBT together with the theoretical potentials of hydrogen evolution and water oxidation; (c) steady-state PL emission spectra of COF-BBT, COP-BBT and the model molecule under 400-nm excitation; (d) EPR spectra of COF-BBT and COP-BBT under visible-light irradiation (λ > 420 nm); (e) TA spectra of COF-BBT at different decay times under 525-nm excitation; (f) TA traces of ESA signals from COF-BBT and COP-BBT probed at 560 and 625 nm, respectively.

The optical properties and electronic structures of our samples were next investigated. The ultraviolet-visible (UV–Vis) diffuse reflectance spectra of COF-BBT and COP-BBT have an adsorption edge at ∼700 nm and a tail extended up to ∼800 nm (Fig. 3a). Corresponding Tauc plot analysis suggests a narrow optical band gap of ∼2.0 eV for both of them (Supplementary Fig. S14). Mott-Schottky analysis of COF-BBT reveals that its lowest unoccupied molecular orbital (LUMO) locates at –1.02 V versus normal hydrogen electrode at pH 7 (Supplementary Fig. S15). The highest occupied molecular orbital (HOMO) is accordingly determined to be at +1.01 V based on its optical band gap as depicted in Fig. 3b. The band structure of COF-BBT is also confirmed using ultraviolet photoelectron spectroscopy and cyclic voltammogram analyses (Supplementary Figs S16 and S17). Density functional theory (DFT) simulations show that the electron density of HOMO is centered on the BBT unit, whereas that of LUMO mainly distributes over the keto unit (Supplementary Fig. S18). Their uneven distributions imply the possible electron transfer from BBT to keto upon light excitation.

High structural crystallinity is beneficial to the charge separation and transport during photocatalysis. In light of their very similar chemical compositions, surface wettability and optical properties, COF-BBT and COP-BBT here form an ideal couple for studying the effect of structural crystallinity on their charge carrier dynamics. Figure 3c compares their steady-state photoluminescence (PL) emission spectra as well as that of the model molecule. Powdery COF-BBT exhibits significantly suppressed PL emission compared to those of COP-BBT and the model molecule. The average fluorescence lifetimes of COF-BBT and COP-BBT are determined to be 296 and 248 ps, respectively (Supplementary Fig. S19). The electron paramagnetic resonance (EPR) spectrum of COF-BBT exhibits a three-times more intense signal relative to that of COP-BBT, indicative of the enhanced exciton dissociation thanks to its ordered molecular frameworks (Fig. 3d) [28,29]. COF-BBT thin film grown on a fluorine-doped tin oxide electrode also exhibits substantially enhanced photocurrent density in solution compared with COP-BBT and the model molecule (Supplementary Fig. S20).

Moreover, we performed ultrafast transient absorption (TA) spectroscopic measurements to study the dynamics of photo-excited carriers. The transmittance (*T*) rather than other absorbance of the samples was recorded using our experimental set-up. As shown in Fig. 3e and Supplementary Fig. S21, the ground-state bleaching signals (Δ*T*/*T* > 0) in the range of 450−550 nm are observed for both COF-BBT and COP-BBT [30]. Simultaneously, the excited-state absorption (ESA) signals appear in the longer wavelength range upon optical pump, which is likely caused by the primary charge-transfer excitations. Notably, the ESA signal is more pronounced in COF-BBT and persists for a longer timescale than that in amorphous COP-BBT (Fig. 3f and Supplementary Fig. S22). The excited-state lifetime of the former is estimated to be ∼20 ps, which is 3.3 times longer than that of the latter (6 ps). This possibly reflects the fewer structural defects in the ordered framework that effectively retard the charge trapping and recombination [31]. All the above spectroscopic characterizations unambiguously corroborate that the high structural crystallinity of COF-BBT affords it with much enhanced charge carrier dynamics.

### Photocatalytic H_2_ production

**Figure 4.**
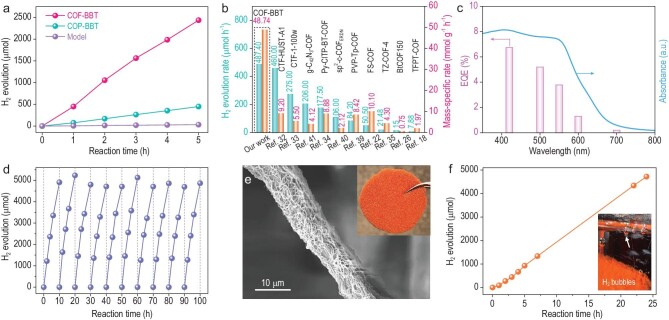
Photocatalytic H_2_ production on COF-BBT. (a) Photocatalytic H_2_ production curves of COF-BBT, COP-BBT and the model molecule; (b) comparison on the photocatalytic H_2_ evolution rate and the mass-specific H_2_ evolution rate of COF-BBT and previous states of the art from literature; (c) wavelength-dependent EQE values of COF-BBT together with its UV–Vis spectra; (d) consecutive cycles of photocatalytic H_2_ production on COF-BBT for ≤100 h; (e) digital image and SEM image of COF-BBT loaded melamine foam; (f) photocatalytic H_2_ production curve of COF-BBT loaded melamine foam; the inset shows H_2_ bubbles evolving from the foam upon the light irradiation.

For the next step, we assessed the photocatalytic performance of COF-BBT for hydrogen production under the illumination of a 300-W Xe lamp equipped with a UV-cut-off filter (>420 nm). As with other COF-based photocatalysis, ascorbic acid was introduced in our experiments as the sacrificial electron donor. An optimized 3 wt% of Pt nanoparticles were *in situ* photo-deposited as the cocatalyst (Supplementary Fig. S23). Supplementary Fig. S24 illustrates the high-resolution TEM image of Pt-deposited COF-BBT. X-ray absorption (XAS) analysis at the Pt L3-edge discloses the non-zero valence state of Pt and the prevailing Pt–O bonding likely resulting from the interaction between Pt and oxygen atoms of the keto nodes (Supplementary Fig. S25). The actual Pt content is measured to be 2.4 wt% by inductively coupled plasma (ICP) analysis. Figure 4a depicts the time-dependent hydrogen evolution curve of COF-BBT (10 mg) for the first 5 h; 450 μmol of H_2_ is produced during the first hour. Its amount accumulates linearly with the reaction time and reaches 2437 μmol at the end of the 5 h of irradiation. This translates into an average hydrogen production rate of 487 μmol h^−1^ or an average mass-specific rate of 48.7 mmol g^−1^ h^−1^—both are among the highest values ever reported for COF-based photocatalysis to date (Fig. 4b and Supplementary Table S1) [32–36]. Worth highlighting is that its mass-specific rate is more than four times larger than the previous COF-based state-of-the-art sulfone-containing 2D-COF developed by Cooper and co-workers [22]. We are aware that higher mass-specific rates were reported for some organic semiconductors beyond COFs, but they were attained by either adopting extremely low catalyst masses or using organic co-solvents [37,38]. Control experiments show that no H_2_ is produced in the absence of light, a photocatalyst or the sacrificial electron donor under otherwise identical conditions. Further, the model molecule has a negligible photocatalytic activity; amorphous COP-BBT was measured to have an average mass-specific H_2_ production rate of 9.0 mmol g^−1^ h^−1^ during the 5 h of photocatalysis. The observed difference in photocatalytic activities is in line with the above analysis of charge carrier dynamics and again highlights the advantage of extended structural crystallinity and surface wettability. We note that once the mesopores of COF-BBT are blocked by polyethylene glycol, its photocatalytic activity significantly decreases (Supplementary Fig. S26). Figure 4c shows the wavelength-dependent external quantum efficiency (EQE) of COF-BBT. A high value of 6.8% is measured at 420 nm, which is superior to most other COF-based photocatalysts available so far (Supplementary Table S1) [39–41]. Impressively, our COF-BBT is photoactive even in the longer wavelength range of the visible spectrum and attains a remarkable EQE value of 0.2% at 700 nm. The EQE value of organic photocatalysts is generally limited (<5%) due to the formation of strongly bound Frenkel excitons [42]. This can be compensated by their broad light absorption. By contrast, inorganic semiconductors (e.g. TiO_2_) and C_3_N_4_ may exhibit higher EQE values in the near-UV region, but usually have negligible absorption at longer wavelengths (>550 nm) that leads to overall lower photocatalytic activities (Supplementary Fig. S27).

Stability of COF-BBT was then evaluated via cycling experiments. As shown in Fig. 4d, there is no activity loss for 10 cycles and a total reaction time of 100 h. This is, however, far from the possible lifetime of our COF-BBT. After the long-term stability test, recollected COF-BBT was subjected to FTIR, UV–Vis, XRD, XPS and TEM characterizations (Supplementary Figs S3 and S28). No obvious structural change is noted, thereby attesting to its great stability. ICP analysis of the solution reveals no discernible increase in the sulfur concentration after photocatalysis, further ruling out possible material degradation (Supplementary Table S2).

We made a few more explorations. Pt cocatalyst here serves as the active site to receive photogenerated electrons from COF-BBT and catalyse hydrogen evolution on the surface. Despite the predominant use of Pt cocatalyst in polymeric photocatalysis, it would be highly tempting to search for non-precious-metal-based alternatives. To this end, we chemically deposited 3 wt% Ni, Co and Cu on COF-BBT (Supplementary Fig. S29) [43]. When evaluated under identical conditions, Ni-loaded COF-BBT is measured to be the most active with an average hydrogen production rate of 7.7 μmol h^−1^ or a mass-specific rate of 770 μmol g^−1^ h^−1^ (Supplementary Fig. S30). Although ∼60 times lower than those of Pt-loaded COF-BBT, this rate is roughly comparable to the state of the art using non-precious-metal-based co-catalysts [44,45].

For practical applications, it is often desired to load powdery photocatalysts onto high-surface-area and photo-inactive supports to facilitate the catalyst recycling [46–48]. We here demonstrate that our COF-BBT can directly grow on porous melamine foams. Successful catalyst loading is reflected from the evident color change of the support from white to orange (Fig. 4e and Supplementary Fig. S31). These catalyst-loaded foams can be readily cut into a variety of shapes and thicknesses as needed for photocatalytic applications. The COF-BBT content is typically controlled to be ∼3 wt%. SEM examination reveals that at such a dilute catalyst concentration, micrometer-long fibers of COF-BBT tightly wrap around the foam skeleton and densely cover the substrate. When immersed into the solution of ascorbic acid and *in situ* photo-deposited with Pt, the catalyst-loaded melamine foam (in a circular shape with 3 cm in diameter and 3 mm in thickness) enables stable H_2_ production under visible-light irradiation, giving rise to a total of 4717 μmol of H_2_ over 24 h (Fig. 4f). This translates into an average H_2_ production rate of 28 μmol cm^−2^ h^−1^. At the end of the reaction, the catalyst can be simply retrieved by taking out the melamine foam from the solution. No visible catalyst falling off is observed and the surface texture of the foam remains (Supplementary Fig. S32).

### Coupling of H_2_ production and the selective oxidation of biomass

Another obstacle in the practical applications of COF-based or, more generally, polymer-based photocatalysts is the common use of sacrificial electron donors [8]. Even though the band structure of COF-BBT is analysed to straddle the potentials of hydrogen evolution and water oxidation, no O_2_ can be measured experimentally in the absence of ascorbic acid with or without co-catalysts (e.g. CoO_x_). This is probably because the water oxidation overpotential (calculated to be ∼190 mV) is too small to effectively drive this demanding half reaction [8]. One solution is to pursue Z-scheme photocatalysis by integrating two different semiconductor materials with proper band offset for separate half reactions [49]. Another strategy is to integrate hydrogen evolution with the oxidation transformation of organic substrates that can take place under milder conditions and yield higher-valued products than O_2_ [50–52].

**Figure 5.**
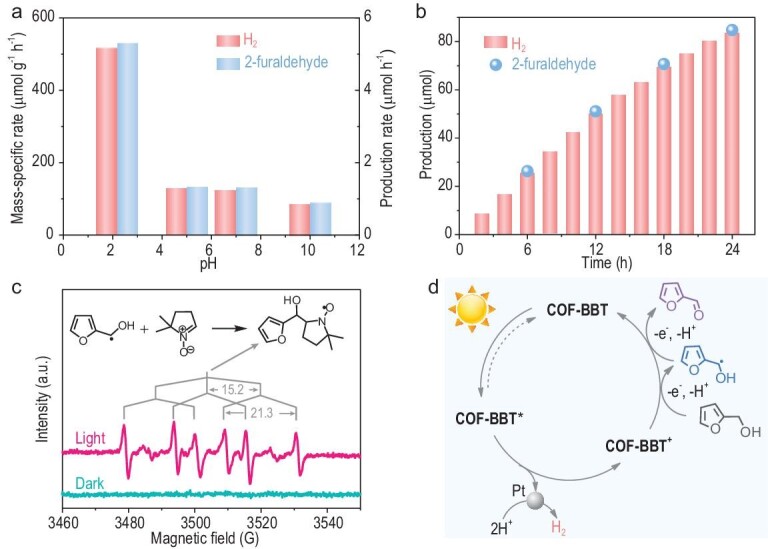
Photocatalytic H_2_ production coupled with the selective oxidation of furfuryl alcohol of COF-BBT. (a) pH-dependent photocatalytic activities for the coupled reaction; (b) photocatalytic co-production of H_2_ and 2-furaldehyde at pH = 2.2 over a long-term reaction period; (c) *in situ* EPR analysis during the photocatalytic oxidation of furfuryl alcohol in the presence of DMPO in the dark or light; (d) proposed reaction mechanism.

In our experiments, we attempted to substitute ascorbic acid with furfuryl alcohol (a common biomass-derived chemical) to scavenge photogenerated holes. During the reaction, furfuryl alcohol is selectively oxidized to 2-furaldehyde, which is an important chemical intermediate for the plastic and pharmaceutical industry [53]. The reaction shows interesting pH dependence (Fig. 5a). The activity is measured to be the highest in pH = 2.2 solution (adjusted with the addition of HClO_4_). The H_2_ and 2-furaldehyde products are found to be stoichiometric and their average mass-specific activities reach 530 μmol g^−1^ h^−1^. Further lowering the solution pH to <2 causes catalyst instability, while increasing the solution pH to >4 noticeably compromises the activity. Control experiments demonstrate the vital roles of different reaction components (Supplementary Fig. S33). Even though the optimal H_2_ production rate measured here is ∼100 times slower than that measured in the presence of ascorbic acid (presumably due to the different hole-scavenging kinetics of furfuryl alcohol and ascorbic acid), the study here suggests that the proposed approach is viable for the first time for polymeric photocatalysts (excluding C_3_N_4_). Future improvement can be made by adjusting the redox potential of the organic substrate and optimizing its interaction with the photocatalyst.

We further investigated the long-term stability of the photocatalytic conversion under the optimal conditions. As shown in Fig. 5b, an approximately linear increase in H_2_ is observed up to 24 h under visible-light irradiation, producing a total amount of ∼84 μmol H_2_. During the reaction, the amount of 2-furaldehyde accumulated in the solution was examined every 6 h and was analysed to be generally consistent with the hydrogen evolution curve. It supports that the two half reactions are close to stoichiometry even over a long reaction period and indicates high reaction selectivity and no over-oxidation of 2-furaldehyde beyond two electrons.

Finally, in order to understand how furfuryl alcohol is oxidized, an *in situ* EPR experiment was carried out during photocatalysis using 5,5-dimethyl-1-pyrroline-N-oxide (DMPO) as the radical trapping agent. No EPR signal was observed in the dark, while a pronounced set of EPR signals were observed in the light, corresponding to the formation of hydroxylmethyl carbon radical during photocatalysis that becomes trapped by DMPO (Fig. 5c) [54,55]. It evidences that the oxidation of furfuryl alcohol to 2-furaldehyde involves a free-radical intermediate. Based on the above results, a schematic cycle mechanism is proposed for photocatalytic hydrogen evolution and furfuryl alcohol oxidation as shown in Fig. 5d.

## CONCLUSION

In summary, we demonstrated BBT-COF as an efficient and robust photocatalyst for hydrogen production. The material featured great structural crystallinity and water wettability owing to the incorporation of rigid and polar BBT units. BBT-COF exhibited fast charge carrier dynamics as evidenced by a range of spectroscopic characterizations and excellent photocatalytic performances when either suspended in solution or supported on melamine foams. Most impressively, a mass-specific hydrogen production rate of 48.7 mmol g^−1^ h^−1^ and EQE of 6.8% at 420 nm were measured in the presence of sacrificial electron donors, which is superior to the previous state of the art. Further, the photocatalytic hydrogen production could be coupled with the oxidation of furfuryl alcohol to 2-furaldehyde at stoichiometry. Our study here highlights the tremendous potential of COF-based photocatalysis to rival or even outperform the so far predominant inorganic-based photocatalysis.

## Supplementary Material

nwac171_Supplemental_File
