# Supplementary material for: Highly crystalline and water-wettable benzobisthiazole-based covalent organic frameworks for enhanced photocatalytic hydrogen production
Source: Natl Sci Rev. 2022 Aug 18;10(1):nwac171. doi: 10.1093/nsr/nwac171 (PMC9843129; doi:10.1093/nsr/nwac171)
Supplement: nwac171_Supplemental_File [file nwac171_supplemental_file.pdf]

Supplementary Data

Highly Crystalline and Water-Wettable Benzobisthiazole-Based Covalent Organic Frameworks for Enhanced Photocatalytic Hydrogen Production

Wei Huang[1,2,ǂ], Yongpan Hu[1,2,ǂ], Zhengyuan Qin[3], Yujin Ji[1,2], Xuan Zhao[1,2], Yunling Wu[1,2], Qing He[1,2], Youyong Li[1,2], Chunfeng Zhang[3], Jun Lu[4,]* and Yanguang Li[1,2,5,]*

[1]Institute of Functional Nano and Soft Materials (FUNSOM), Jiangsu Key Laboratory for Carbon-Based Functional Materials and Devices, Soochow University, Suzhou 215123, China;

[2]Jiangsu Key Laboratory for Advanced Negative Carbon Technologies, Soochow University, Suzhou 215123, China;

[3]National Laboratory of Solid State Microstructures, School of Physics, and Collaborative Innovation Center for Advanced Microstructures, Nanjing University, Nanjing 210093, China;

[4]College of Chemical and Biological Engineering, Zhejiang University, Hangzhou 310027, China

[5]Macao Institute of Materials Science and Engineering (MIMSE), MUST-SUDA Joint Research Center for Advanced Functional Materials, Macau University of Science and Technology, Macau, China;

***Corresponding authors.** E-mails: yanguang@suda.edu.cn; junzoelu@zju.edu.cn

[ǂ]Equally contributed to this work.

Materials:

All chemicals and solvents were purchased from commercial sources and used as received unless otherwise noted.

Characterizations:

Liquid ^{1}H and ^{13}C NMR spectra for all the compounds were measured using a 300 MHz Bruker Avance spectrometer. Solid state ^{13}C CP-MAS NMR measurements were conducted on a 400 MHz Bruker Avance Ⅲ solid state NMR spectrometer equipped with a standard 4 mm MAS double resonance probe head. XRD was performed on a PANalytical X-ray diffractometer with monochromatic Cu Kα radiation. XPS results were obtained on an Ultra DLD X-ray photoelectron spectrometer. UPS analysis was performed on an Ultra DLD X-ray photoelectron spectrometer with a helium discharge lamp using the He I (21.22 eV) radiation line. XAS spectra were collected in the transmission mode at beamline 11B of the Shanghai Synchrotron Radiation Facility (SSRF, China). The spectrum of a Pt foil was used to calibrate the Pt L-edge energy. Solid-state UV-Vis diffuse reflectance spectra were measured on a Perkin Elmer Lambda 950 spectrophotometer. Steady state fluorescence spectra were recorded on a Perkin Elmer Lambda 100 spectrophotometer. SEM images were taken on a Supera 55 Zeiss scanning electron microscope. TEM images were collected on a Talos 200X transmission electron microscope. N_2 sorption experiments were carried out using a Micrometrics ASAP 2020 HD88 analyzer. Samples were degassed for 12 h at 120 °C under vacuum prior to analysis. Water adsorption isotherms were collected on a Micrometrics ASAP 2020 HD88 analyzer at room temperature using the dynamic vapor sorption method where the water uptake was determined gravimetrically. The samples were first degassed at 150℃ under N_2 for 6 h to remove the pre-adsorbed moisture. Normal EPR spectra were measured on a Bruker A300 spectrometer under the light irradiation (λ>420 nm) at room temperature. *In-situ* EPR was performed on a Bruker EMXplus spectrometer with 1 mg of COF-BBT dispersed in 1 mL of furfuryl alcohol and H_2O (9:1,

v:v) in the presence of 0.1 M DMPO. Signals were collected in the dark or after the light irradiation for 5 min. The water contact angle measurements were conducted on thin film samples at room temperature using a Dataphysics OCA 15 plus contact angle goniometer. TGA was conducted on a Mettler Toledo TGA/DSC1 thermal analyzer under N_2 with the temperature ramped from 25 °C to 800 °C at a rate of 10 °C/min. DSC was conducted on a TA DSC 2010 differential scanning calorimeter up to 300 ℃ under N_2 at a heating rate of 10 ℃ /min.

Material synthesis:

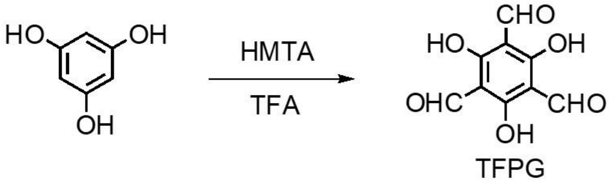

Synthesis of triformylphloroglucinol (TFPG): It was synthesized according to the literature (*J. Am. Chem. Soc.* **2012**, *134*, 19524-19527). 15.1 g of hexamethylenetetramine (HMTA) (108 mmol) and 6.0 g of dried phloroglucinol (49 mmol) were first added to 90 mL of trifluoroacetic acid (TFA) under N_2. The solution was heated at 100 ℃ for 2.5 h. It was then added with ~150 mL of 3 M HCl solution, and further heated at 100 ℃ for 1 h. After cooling down to room temperature, the solution was filtered through Celite. The filtrate was extracted with dichloromethane (200 mL) twice. The organic phase was collected and washed with brine and dried over anhydrous magnesium sulfate. After filtration, the solvent was removed by rotary evaporation to give rise to the titled product as off-white powders. Yield: 16%; ^{1}H NMR (300 MHz, $CDCl_3$) δ 14.10 (s, 3H, OH), 10.14 (s, 3H, CHO); ^{13}C NMR δ 162.1, 173.6, 102.9.

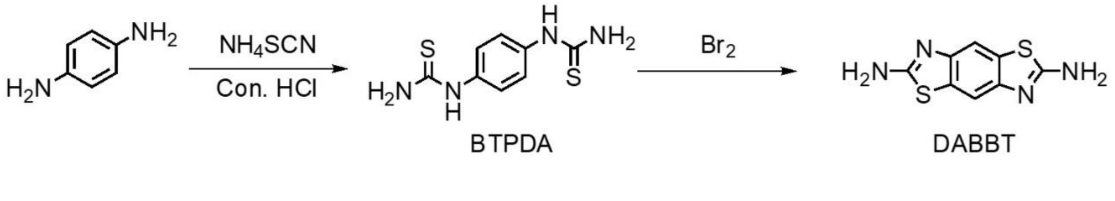

Synthesis of N,N'-bis(thiocarbamoyl)-1,4-phenylenediamine (BTPDA): It was synthesized according to the literature (*J. Mater. Chem. C,* **2017**, *5*, 11927-11936). 10.8 g of 1,4-phenylenediamine (0.1 mol), 20 mL of concentrated HCl (38 wt%) solution and 1 g of activated carbon were added into 90 mL of distilled H_2O. The mixture was heated to 50 ℃ and stirred for 30 min under N_2, followed by filtration to remove the activated carbon. The filtrate was then added with 31 g of ammonium thiocyanate (0.4 mol) and heated at 90 ℃ under magnetic stirring and N_2 protection for 12 h. After cooled down to room temperature, the yellow solid was collected by filtration, thoroughly washed with hot H_2O and finally dried under vacuum at 80 ℃ overnight. Yield: 92 %; ^{1}H NMR (300 MHz, DMSO-d6) δ 9.61 (s, 2H), 7.29 (s, 4H, Ar); ^{13}C NMR δ 181.4, 135.9, 124.2.

Synthesis of 2,6-diaminebenzobisthiazole (DABBT): It was synthesized according to the literature (*J. Mater. Chem. C,* **2017**, *5*, 11927-11936). 20 g of BTPDA (88.5 mmol) was first dissolved in 90 mL of dry $CHCl_3$ under vigorous stirring, to which 32.7 g of Br_2 (204.4 mmol) in 20 mL of dry $CHCl_3$ was added dropwise while carefully maintaining the solution temperature below 50 ℃. The orange suspension was further stirred at room temperature overnight and subsequently refluxed at 70 ℃ for another 24 h. After cooling down to room temperature, the orange solid was collected by filtration, washed with CH_3Cl and dried in air. Next, the crude product was dispersed in 100 mL of 10 wt% $NaHSO_3$ aqueous solution for 30 min, followed by filtration and washing with 2 M ammonia aqueous solution and distilled H_2O. The solid powders were then dissolved in 150 mL of 1 M HCl. After filtration, the pH value of the filtrate was adjusted to 10 with concentrated ammonia solution (38 wt%). The resultant pale slurry was collected by centrifugation, washed with H_2O and dried under vacuum at 80 ℃ for 24 h. Finally, the product was purified via the recrystallization in glacial acetic acid. Yield: 46%; ^{1}H NMR (300 MHz, DMSO-d6) δ 7.61 (s, 2H, Ar), 7.32 (s, 4H, NH_2); ^{13}C NMR δ 165.4, 140.8, 129.5, 110.0.

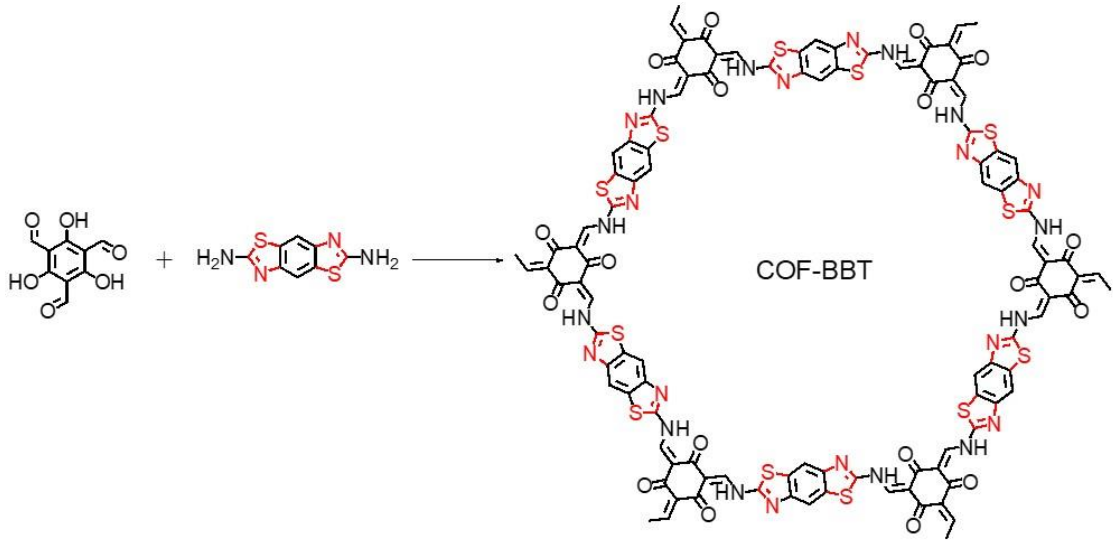

Synthesis of crystalline COF-BBT: Typically, 1.05 g of TFPG (5 mmol) and 1.67 g of DABBT (7.5 mmol) were ultrasonically dispersed in a mixture of *o*-dichlorobenzene, *n*-butyl alcohol and 6 M acetic acid (100 ml, 5/5/1, v/v/v) contained in a 200 mL ventilation vial. The vial was then placed into an electric oven and heated at 120 ℃ for 72 h. The solid product was collected via filtration, thoroughly washed with *N*, *N*-dimethylformamide (DMF), tetrahydrofuran (THF) and finally vacuum-dried at 120 ℃ overnight with a typical yield of 2.24 g. Yield: 92%.

Preparation of melamine foam-supported COF-BBT: 10.5 mg of TFPG (0.05 mmol) and 16.8 mg of DABBT (0.08 mmol) were first ultrasonically dispersed in a mixture of *o*-dichlorobenzene, *n*-butyl alcohol and 6 M acetic acid (10 mL, 5/5/1, v/v/v) contained in a 100 mL ventilation vial. Subsequently, a piece of melamine foam with a diameter of 40 mm and thickness of 3 mm was immersed into the above solution. The solution was sealed and heated at 120 ℃ for 72 h. After cooling down to room temperature, the foam was taken out from the mother liquid, ultrasonically washed with DMF and THF, and finally dried at 120 ℃ overnight under vacuum.

Synthesis of amorphous COP-BBT: The amorphous COP-BBT was synthesized via the direct polycondensation of 42 mg of TFPG (0.2 mmol) and 66.6 mg of DABBT (0.3 mmol) in 10 mL of DMF at 120 ℃ for 24 h. The resultant product was filtrated and washed with DMF, THF and finally vacuum-dried at 120 ℃ overnight to give 84 mg of the titled product. Yield: 86%.

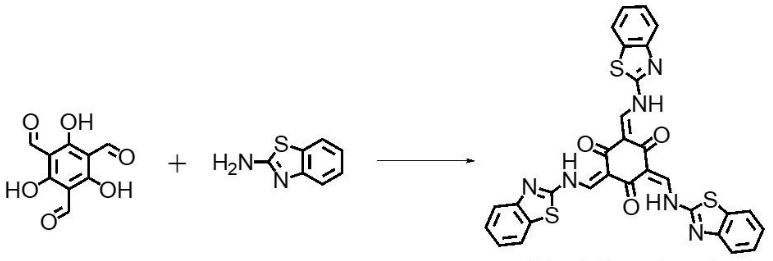

Synthesis of the model molecule: 42 mg of TFPG (0.2 mmol) and 180 mg of 2-amine-benzothiazol (1.2 mmol) were dispersed in 80 mL of anhydrous ethanol. The dispersion was heated at 85 ℃ under N_2 for 24 h. Solid powders were collected by filtration, washed with ethanol, and finally dried at 80 ℃ overnight under vacuum to give 201 mg of yellow powder. Yield: 95 %.

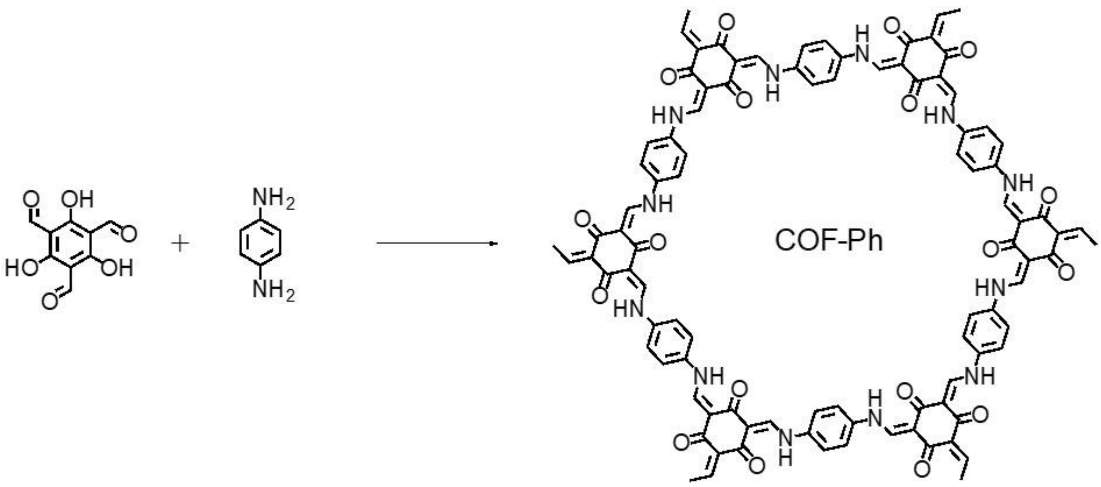

Synthesis of COF-Ph: COF-Ph was synthesized according to the literature (*J. Am. Chem. Soc.* **2012**, *134*, 19524-19527). Typically, a 25 mL Pyrex tube was charged with 63 mg of TPTG (0.3 mmol), 48 mg of *o*-diaminobenzene (0.45 mmol), 1.5 mL of mesitylene, 1.5 mL of dioxane and 0.5 mL of 0.3 M acetic acid aqueous solution. It was sonicated for 10 min, bubbled with N_2 for 10 min, and then sealed and heated at 120 ℃ for 72 h. The resultant solid powders were collected by centrifugation, washed with THF and acetone, and finally dried at 120 ℃ for 12 h under vacuum. Yield: 83%.

Photocatalytic measurements

Photocatalytic hydrogen production was evaluated in an online photocatalytic system (Solarlab-6A, Perfectlight). Typically, 10 mg of the photocatalyst powder under investigation was dispersed in 50 mL of 0.1 M ascorbic acid solution contained in a top-irradiation Pyrex vessel. The dispersion was ultrasonicated for 1 h. A calculated volume of 0.01 mM H_2PtCl_6 aqueous solution (corresponding to ~3 wt% Pt relative to the photocatalyst) was introduced to the reaction solution for the *in-situ* photodeposition of Pt nanoparticles as the cocatalyst. The solution was then carefully evacuated three times to remove the air and filled with Ar. During photocatalysis, the solution was irradiated by a 300 W Xe-lamp with a cutoff filter (λ > 420 nm). Its temperature was maintained at 20 ℃ by water circulation through the external jacket. The amount of H_2 was measured by an online gas chromatograph (SHIMADZU 2010) equipped with a TCD detector and using the Ar carrier gas.

In order to couple the hydrogen production with the oxidation conversion of furfuryl alcohol, the reaction was conducted in 50 mL of 0.1 M furfuryl alcohol aqueous solution with its pH adjusted to different values using 0.1 M $HClO_4$ or 0.1 M NaOH. The oxidation product − 2-furaldehyde was measured using a high-performance liquid chromatograph (HPLC, Ultimate 3000) equipped with an UV-Vis detector set at 220 nm and a Shim-pack GWS C18 column (L: 150 mm, I.D.: 4.6 mm, 5 μm). Its mobile phase was composed of CH_3CN and H_2O with a volume ratio of 1:1. The flow rate was set at 1 mL/min. The content of 2-furaldehyde was quantified according to the external calibration plot.

EQE Measurements

For EQE measurements, a monochromatic light (λ = 420, 500, 550, 600 and 700 nm) from a Xe-lamp was used as the light source, and its intensity was adjusted to be 1.2 mW cm^{-2} using attenuation filters. 10 mg of COF-BBT was dispersed in 20 mL of 0.1 M ascorbic acid and H_2PtCl_6 solution (Pt ~3 wt% relative to COF-BBT). The illumination area was 12 cm^2. The EQE value was calculated using the following equation:

$$EQE\% = 2 \times (n \cdot N_A \cdot h \cdot c) / (I \cdot S \cdot t \cdot \lambda) \times 100\%$$

where n was the amount of hydrogen detected, N_A was the Avogadro constant (6.022×10^{23} mol^{-1}), h was the Planck constant (6.626×10^{-34} J s), c was the speed of light (3×10^{8} m s^{-1}), S was the irradiation area, I was the light intensity, t was the photoreaction time and λ was the wavelength of the monochromatic light.

Photoelectrochemical (PEC) and Mott-Schottky measurements:

The working electrodes were fabricated by directly growing polymer thin films on FTO conducting glass. An exposed area of 0.5×0.5 cm^2 was defined by epoxy resin coating. A standard three-electrode system consisting of a catalyst-deposited FTO electrode as the working electrode, a Pt wire as the counter electrode, and a SCE as the reference electrode was adopted. PEC was carried out in 0.2 M Na_2SO_4 aqueous solution. The working electrode was biased at 0 V versus SCE and irradiated from the back side by a 300W xenon lamp equipped with a 420 nm cut-off filter. The Mott-Schottky analysis was conducted in the dark at the frequency of 1000 and 1500 Hz using a Gamry Reference 600 potentiostat.

Transient absorption spectroscopy (TAS) measurements:

TAS was obtained using a home-built system. The pump and probe pulses were generated by a Ti:Sapphire regenerative amplifier (Libra, Coherent) at 800 nm with a repetition rate of 1 kHz and pulse duration of 90 fs. A portion of 800 nm output beam was employed to generate the pump beam at 525 nm by an optical parametric amplifier (OperA Solo, Coherent). Another portion of the femtosecond laser beam was focused onto a sapphire plate to generate a broadband supercontinuum light source as a probe beam. The chirp of the probe supercontinuum was corrected with error to be less than 100 fs over the whole spectral range. The time delay between the pump and probe pulse was enabled by a translation stage. The TAS signal was analyzed in terms of differential transmittance ($\Delta T/T$) by a silicon CCD (S3070-1006 Hamamatsu) with a monochromator (Acton

2358 Princeton Instrument) at 1 kHz enabled by a custom-built control board from Entwicklungsbuero Stressing.

Structural simulations:

The Vienna ab initio simulation package (VASP) with a plane-wave basis set (*Phys. Rev. B* **1996**, *54*, 11169-11186) was adopted to optimize the atomic positions and lattice constants of COF crystals having the A-A or A-B stacking mode. The electronic cut-off energy was set to be 520 eV. The Perdew-Burke-Ernzerhof (PBE) formula were used to describe the exchange-correlation energy (*Phys. Rev. Lett.* **1996**, *77*, 3865). The weak van der Waals interaction was corrected by the empirical Grimme DFT-D3 method (*J. Chem. Phys.* **2010**, *132*, 154104). A Gamma-centered Monkhorst-Pack K point mesh of $4 \times 4 \times 40$ was sampled in the first Brillouin zone (*Phys. Rev. B* **1976**, *13*, 5188-5192). Rietveld refinement was performed to further modify the geometry file. HOMO-LUMO distribution was calculated based on density functional theory (DFT) at the B3LYP/6-31G (d) level in Gaussian 16 program.

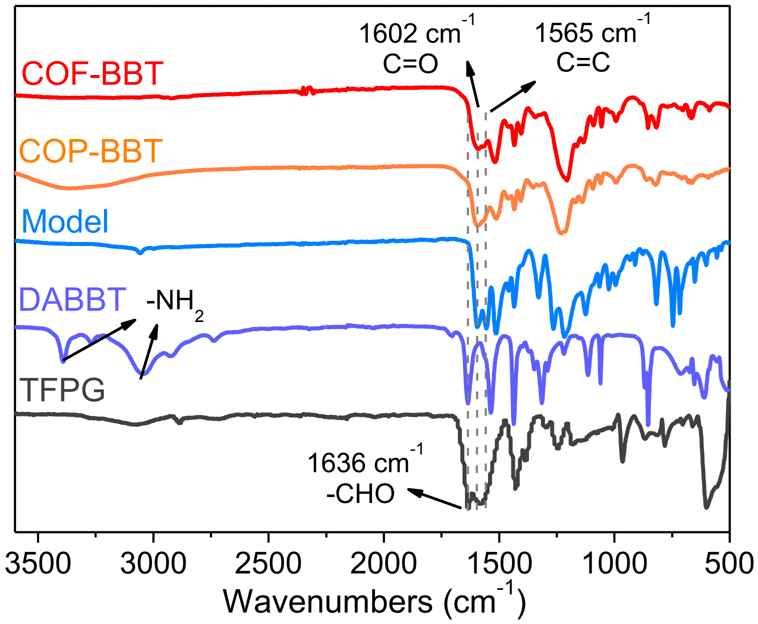

Figure S1. Fourier-transform infrared (FTIR) spectra of crystalline COF-BBT, amorphous COP-BBT, the model molecule, DABBT and TFPG.

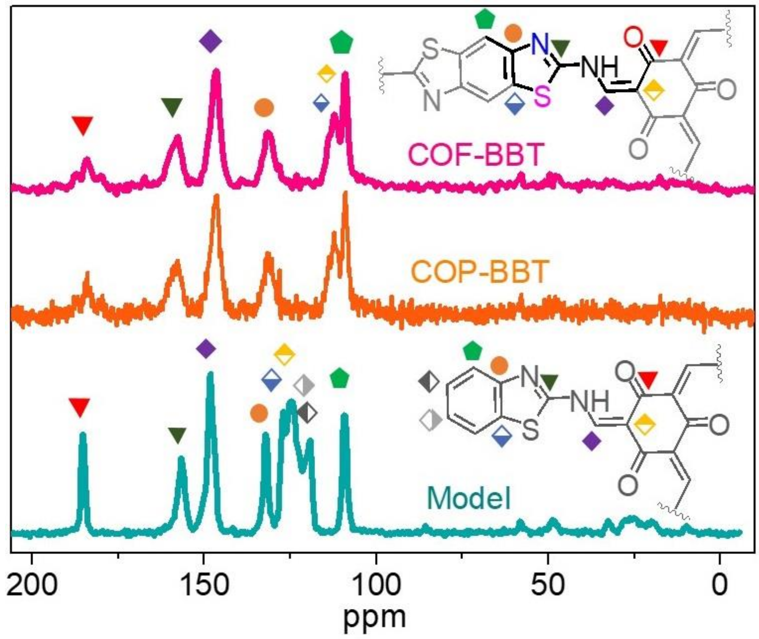

Figure S2. Solid-state NMR spectra of COF-BBT, COP-BBT and the model molecule. The spectra of COF-BBT and COP-BBT reveal the presence of the ketone carbon at 184 ppm. The peak at ca. 160 ppm is assignable to the sp^2 imine carbon (>C=N-) of the BBT moiety, and thereby confirms its incorporation in the molecular frameworks.

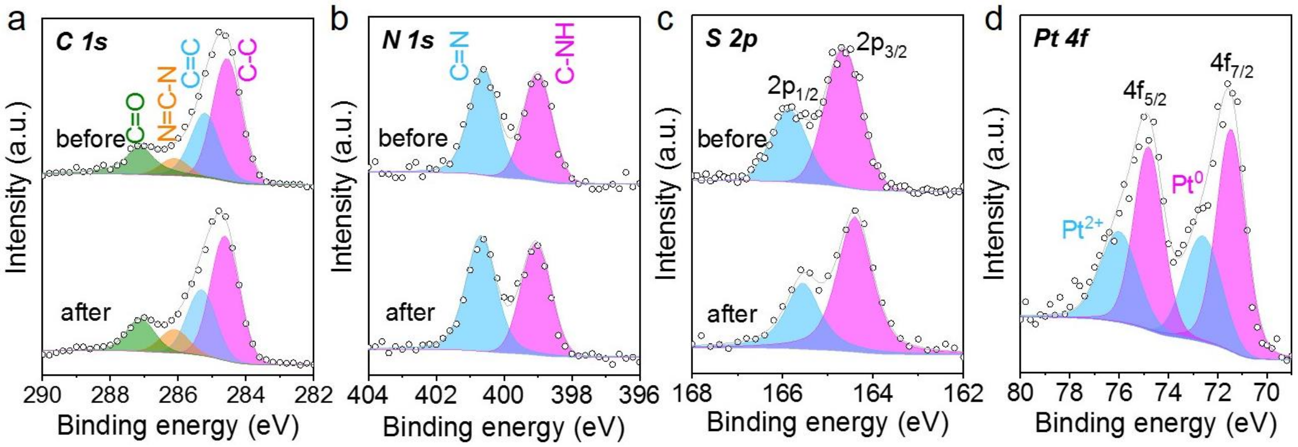

Figure S3. (a) C 1s, (b) N 1s and (c) S 2p XPS spectra of COF-BBT before and after photocatalytic recycling tests; (d) Pt 4f XPS spectrum of COF-BBT after photocatalysis. The C 1s spectrum of COF-BBT can be deconvoluted into four peaks: the ones centered at 284.5 eV and 286.1 eV are respectively contributed by the C=C and C=N bonding in the BBT units; the ones at 285.2 eV and 287.1 eV can be assigned to the C=C and C=O bonding in the keto nodes (*J. Am. Chem. Soc*. **2012**, *134*, 19524). The N 1s spectrum of COF-BBT exhibits two intensive peaks at 399.0 eV and 400.6 eV that can be ascribed to the C-NH bond in the enamine and the C=N bond in the BBT units, respectively (*Nat. Commun.* **2019**, *10*, 2467). The doublet at 163-167 eV in the S 2p XPS spectrum corresponds to the sulfur in the thiazole groups (*J. Am. Chem. Soc.* **2019**, *141*, 11082). In addition, the Pt 4f XPS spectrum reveals the existence of both metallic Pt and Pt^{2+} (*J. Am. Chem. Soc.* **2021**, *143*, 16512).

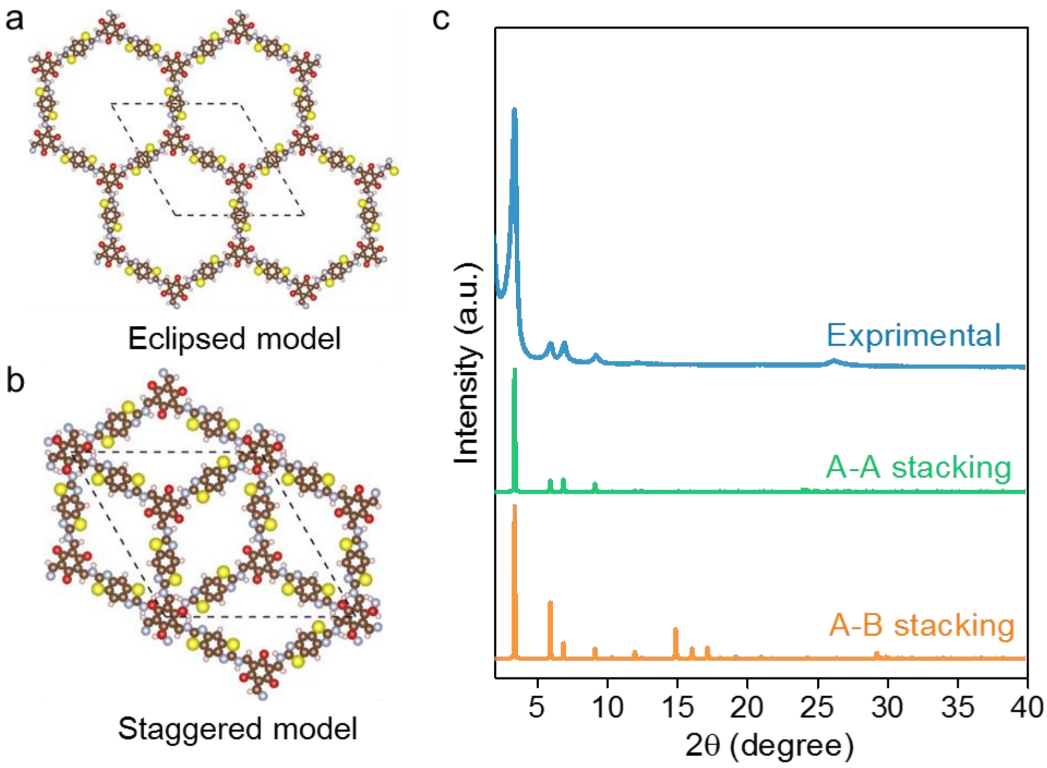

Figure S4. Optimized crystal structure of COF-BBT in (a) the eclipsed (A-A) stacking mode and (b) the staggered (A-B) stacking mode; (c) experimental XRD spectrum compares with the simulated ones based on A-A and A-B stacking.

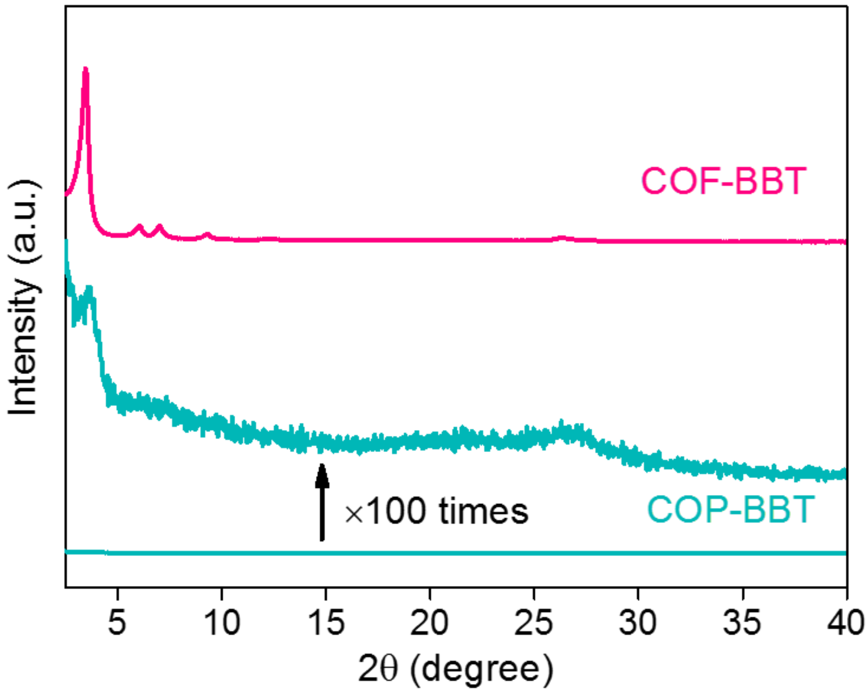

Figure S5. Comparison of XRD spectra of COF-BBT, COP-BBT as well as the magnified spectrum of COP-BBT.

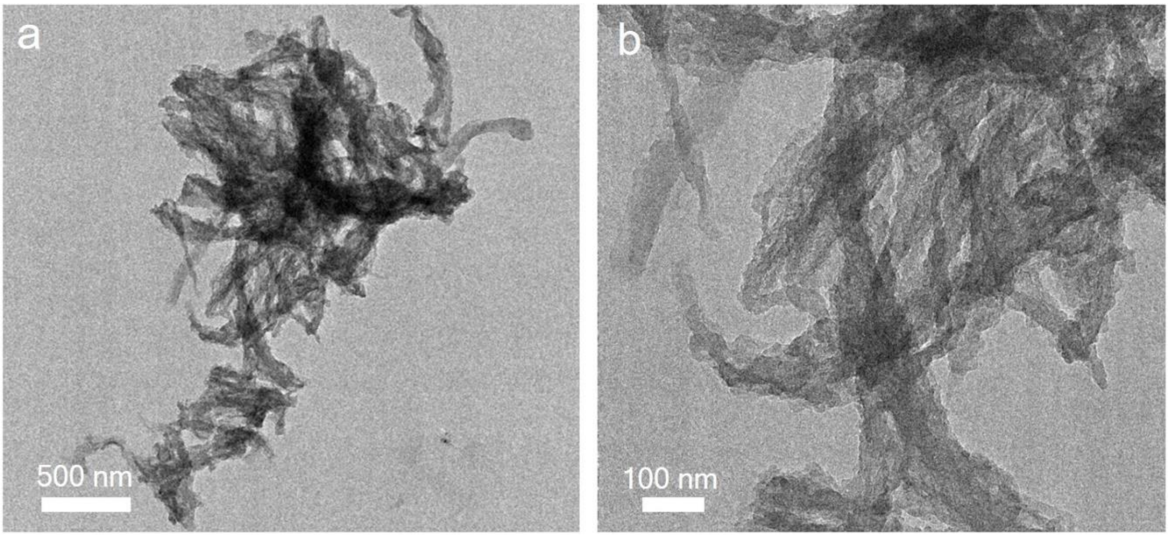

Figure S6. TEM images of amorphous COP-BBT.

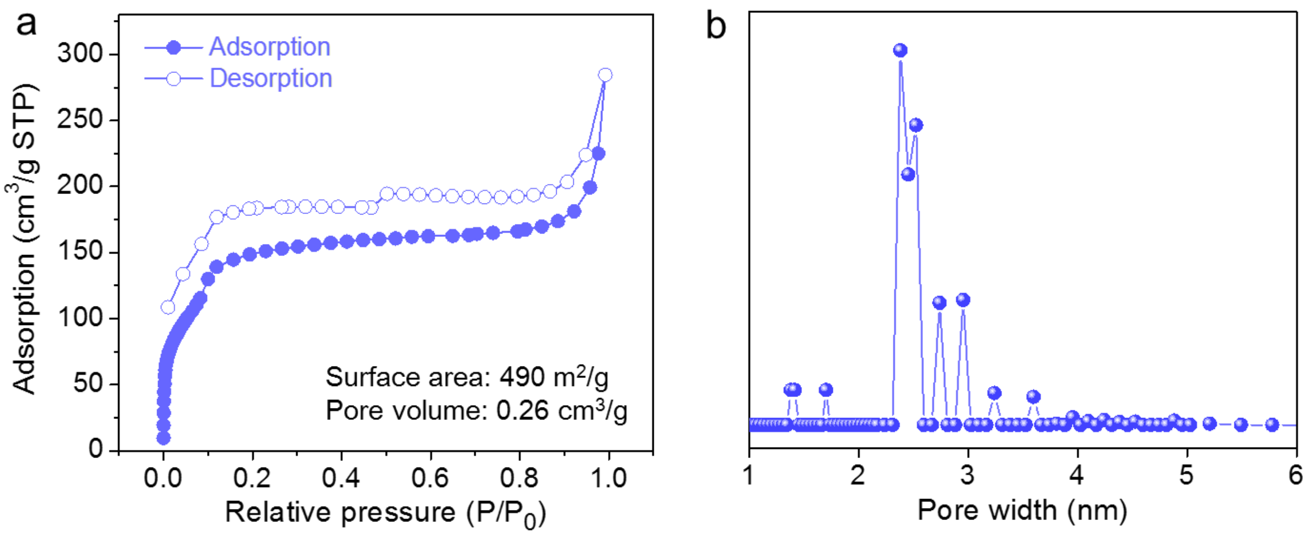

Figure S7. (a) N_2 sorption isotherm and (b) corresponding pore size distribution curve of COP-BBT.

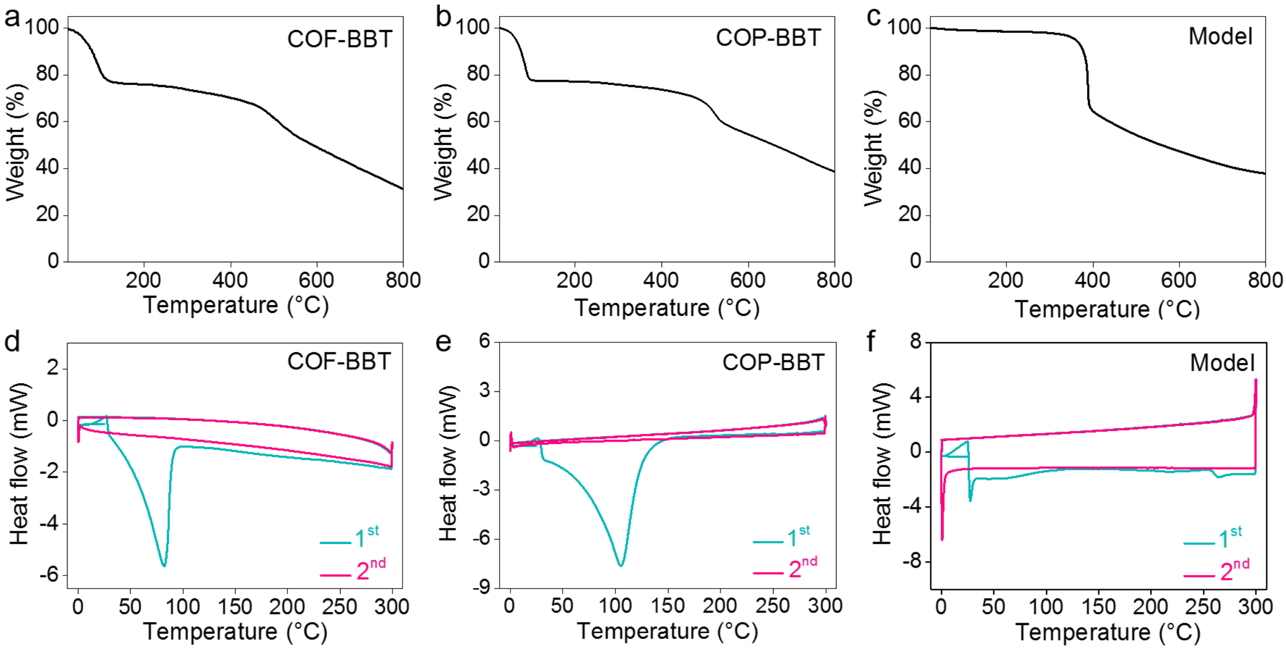

Figure S8. (a-c) TGA curves and (d-e) DSC profiles of COF-BBT, COP-BBT and the model molecule. COF-BBT and COP-BBT have great thermal stability up to 470 ℃ while the model molecule is stable below 400 ℃. The weight loss of COF-BBT and COP-BBT at ~100 ℃ is probably due to the removal of the absorbed water and other gases within the mesopores. Correspondingly, COF-BBT and COP-BBT exhibit a single endothermic peak at ~100 ℃ during the first heating scan, which disappears in the second scan. This result is in line with the TGA observations. No apparent endothermic peak is measured for the model compound probably due to its nonporous nature. For all the three samples, no noticeable endothermic signal is observed that can be associated with the glass transition or melting of the organic frameworks.

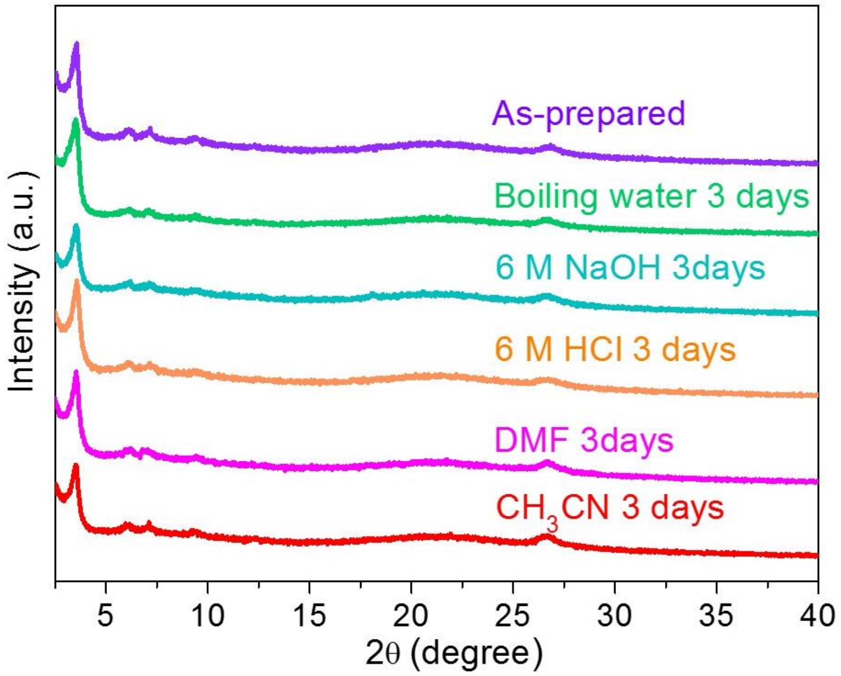

Figure S9. XRD patterns of as-prepared COF-BBT and after treatments in different chemical environments as noted.

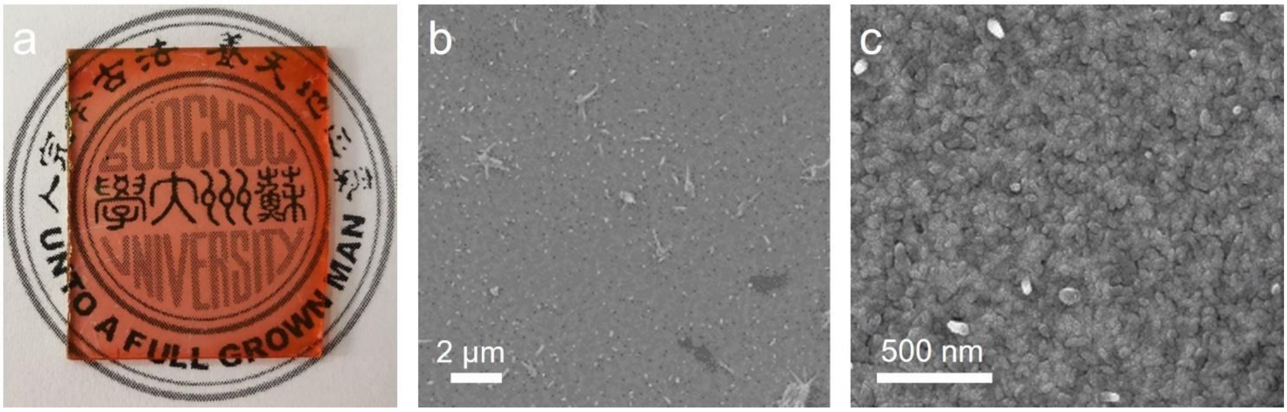

Figure S10. (a) Photograph of a COF-BBT thin film grown on the glass slide and (b, c) its SEM images showing a relatively smooth and dense surface.

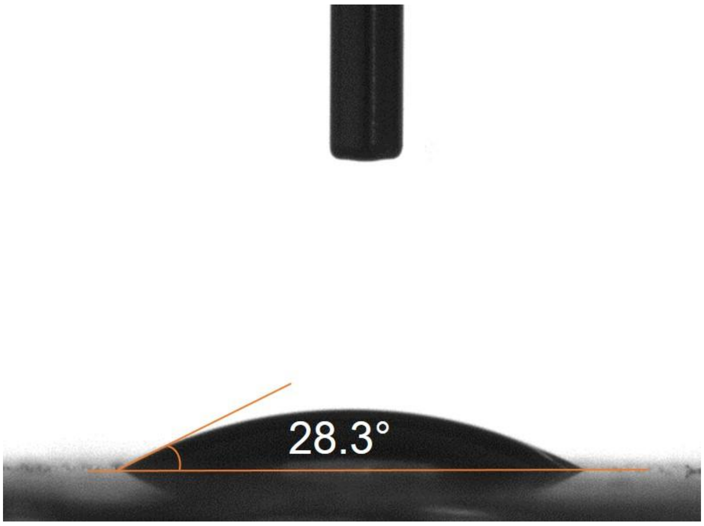

Figure S11. Water contact angle measurement of COP-BBT.

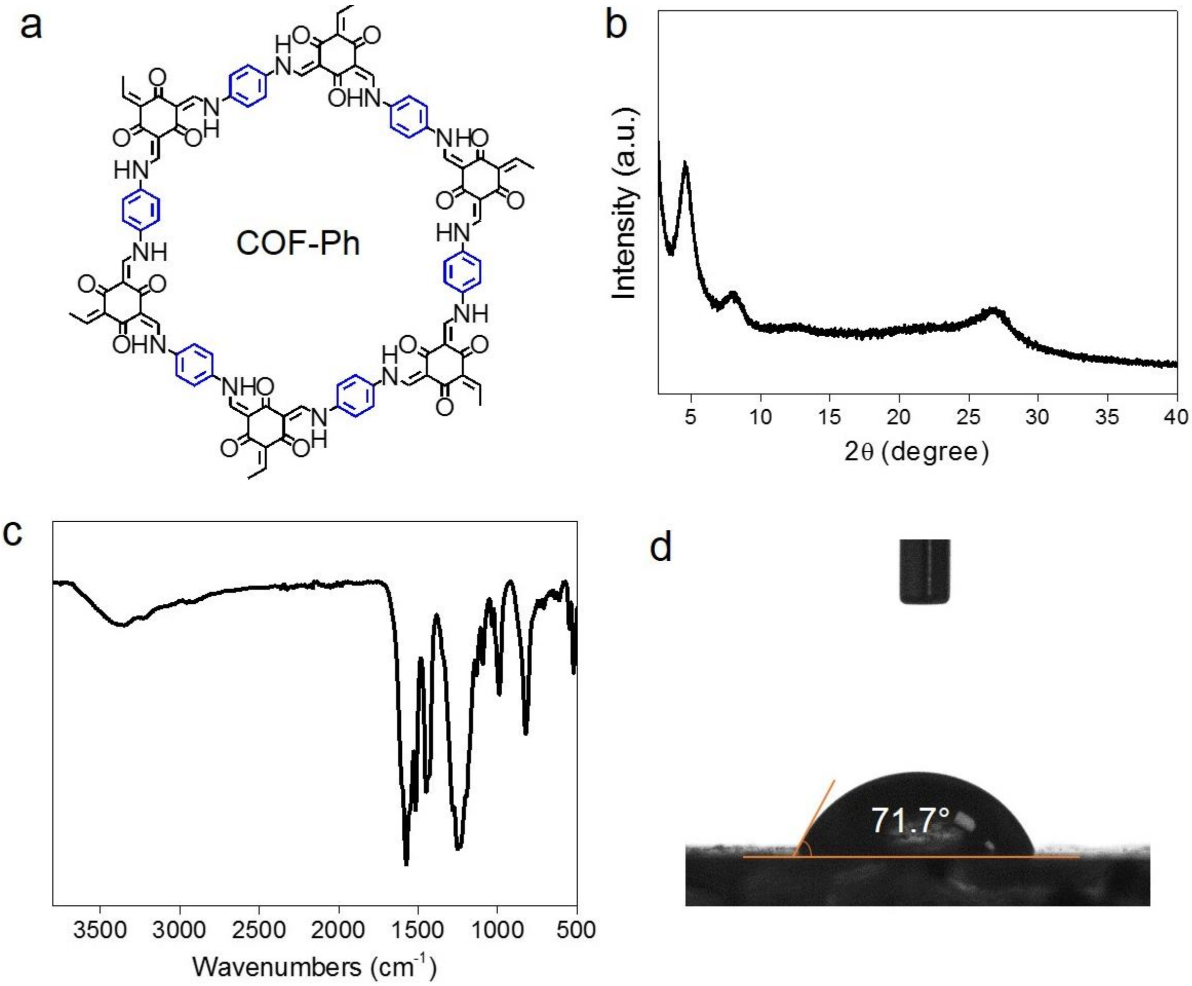

Figure S12. (a) Molecular structure, (b) XRD pattern, (c) FTIR spectrum and (d) water contact angle measurement of COF-Ph.

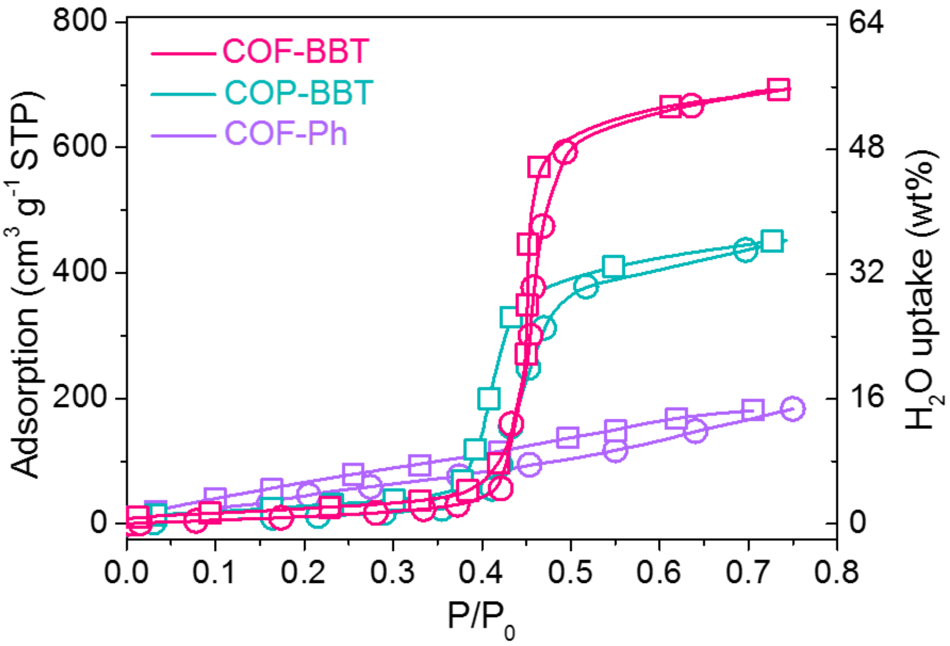

Figure S13. Water sorption isotherms of COF-BBT, COP-BBT and COF-Ph. COP-Ph displays a type-Ⅶ water sorption isotherm, evidencing its relatively hydrophobic nature. COF-BBT and COP-BBT show type-Ⅴ water sorption isotherms that are typically observed for hydrophilic porous sorbents (*Micropor. Mesopor. Mater.* **2008**, *114*, 1). The greater water sorption capability of COF-BBT than COP-BBT is also consistent with the larger surface area of the former from N_2 sorption analysis.

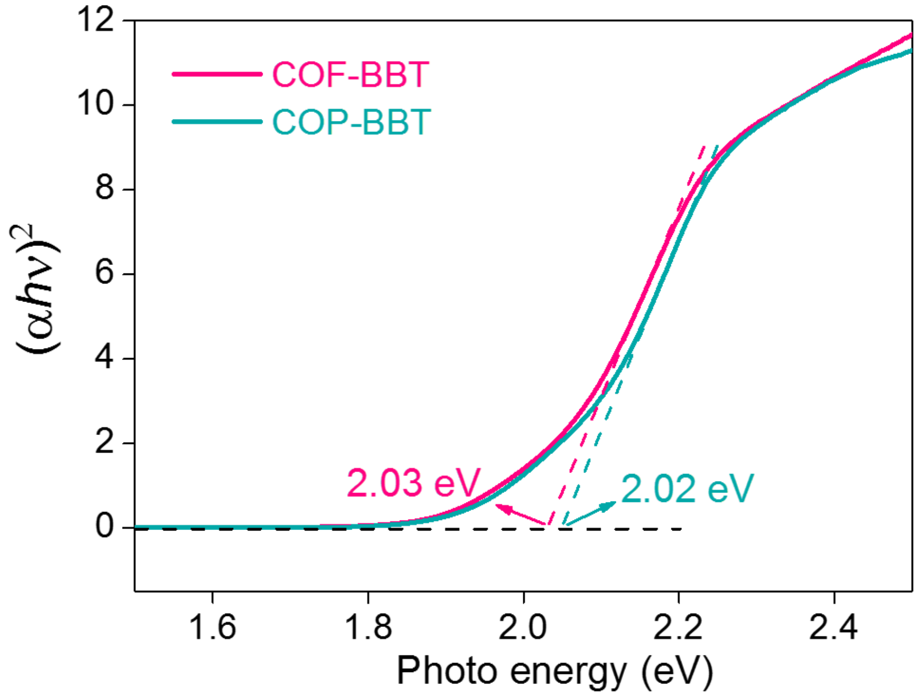

Figure S14. Tauc plots of COF-BBT and COP-BBT.

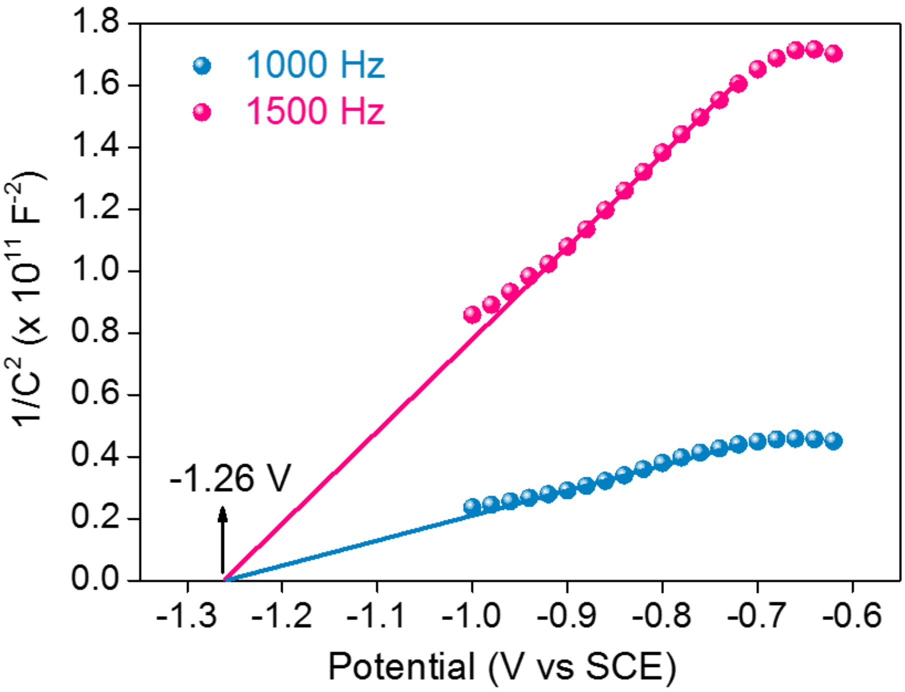

Figure S15. Mott-Schottky plots of COF-BBT in 0.2 M Na_2SO_4.

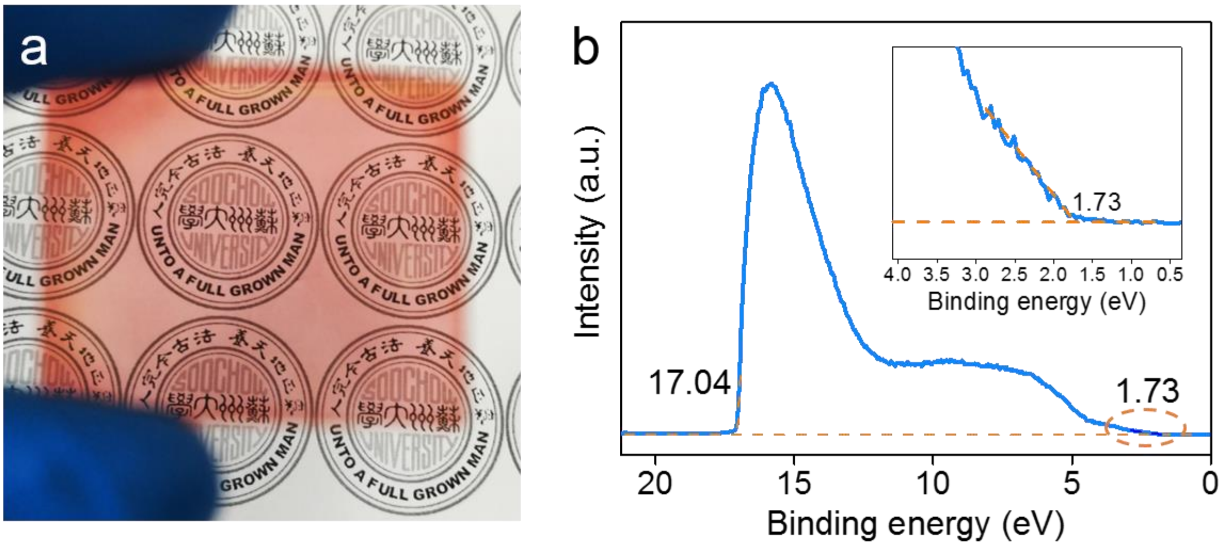

Figure S16. (a) Photograph of the uniform COF-BBT film directly grown on FTO glass and (b) its UPS spectrum. The dashed lines in the UPS spectrum mark the baseline and the tangents of the curve. From the spectrum, the valence band of COF-BBT is determined to be -5.91 eV vs. the vacuum level (17.04 eV – 1.73 eV – 21.22 eV = -5.91 eV), corresponding to 1.05 V vs. NHE in pH = 7 solution (5.91 V - 4.44 V – 7 × 59 mV = 1.05 V, assuming the vacuum level corresponding to -4.44 V vs. NHE).

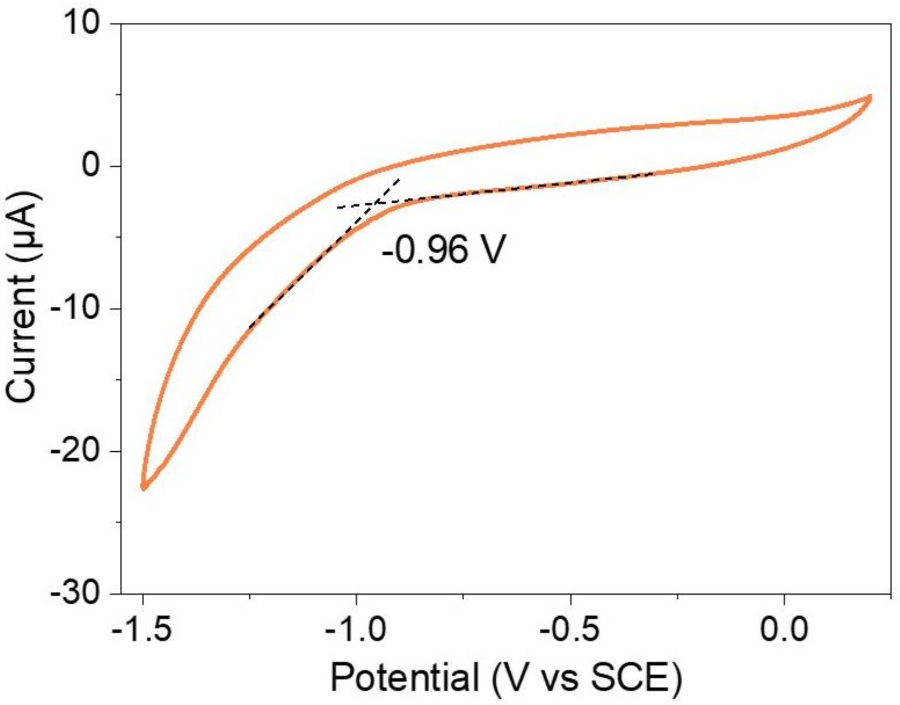

Figure S17. CV curve of COF-BBT in the anhydrous acetonitrile solution of 0.1 M NBu_4PF_6 at a scan rate of 20 mV s^{-1}. From the onset potential of the reduction wave, the LUMO of COF-BBT can be derived to be -0.96 V vs. SCE or -0.72 V vs. SHE, which can be translated to -1.14 V vs. NHE in pH 7 solution.

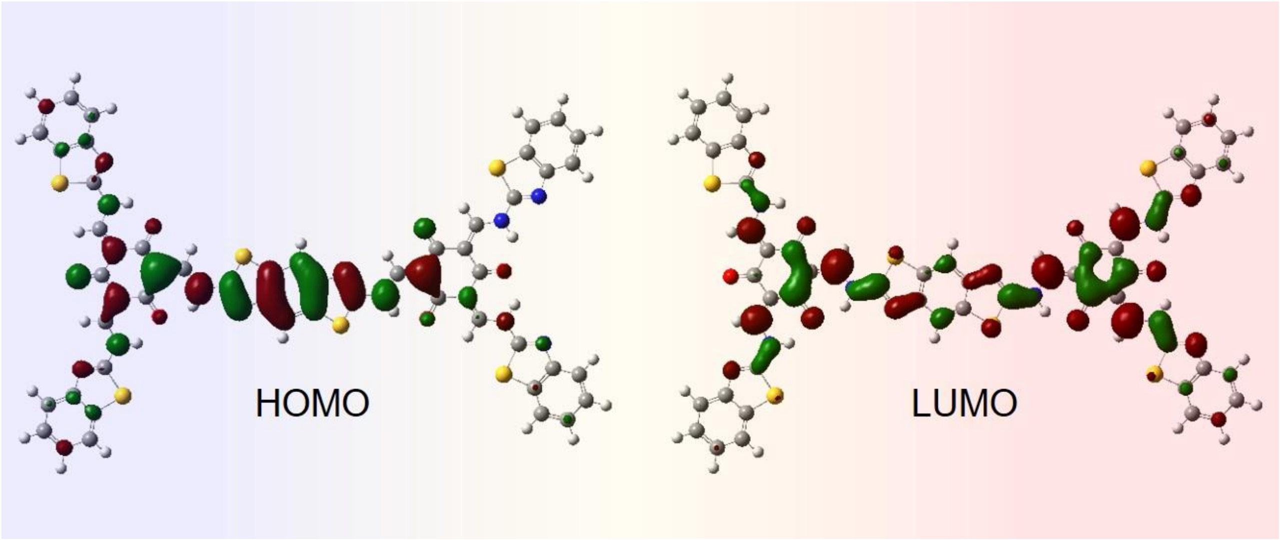

Figure S18. Electron density distribution of COF-BBT HOMO and LUMO simulated using DFT on the level of B3LYP 6-31G (d).

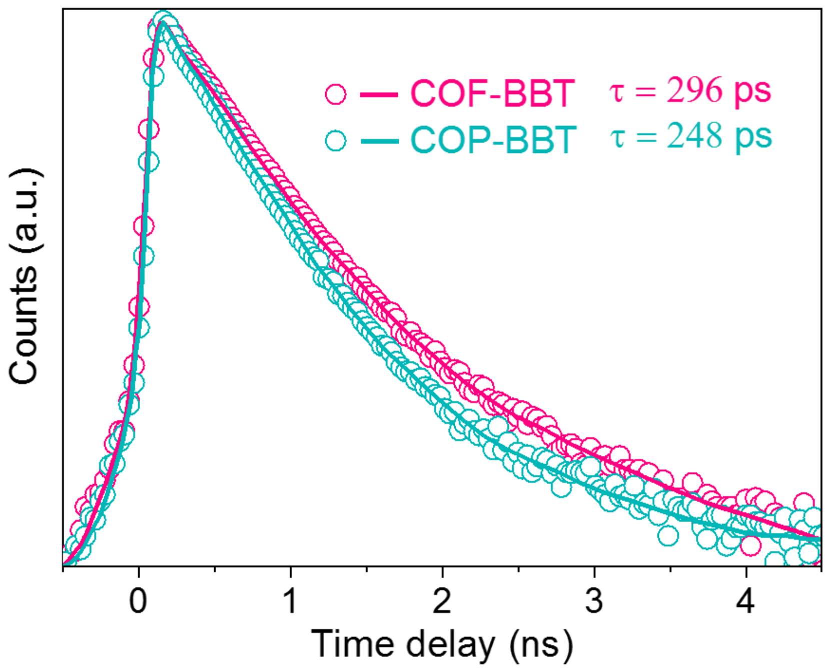

Figure S19. Fluorescence decay curves of COF-BBT and COP-BBT under the 405 nm excitation as well as their fitting results. The fluorescence lifetimes of the thin-film samples are derived by fitting the fluorescence decay curves with triexponential functions.

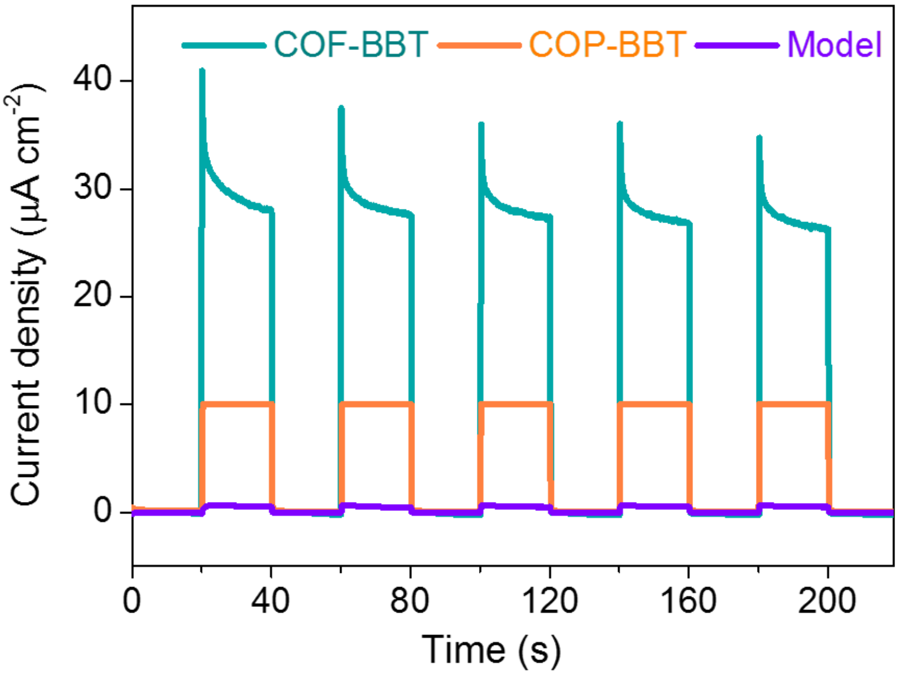

Figure S20. Photocurrent responses of COF-BBT, COP-BBT and the model molecule under the periodic light-off cycles when biased at 0 V vs. SCE in 0.2 M Na_2SO_4.

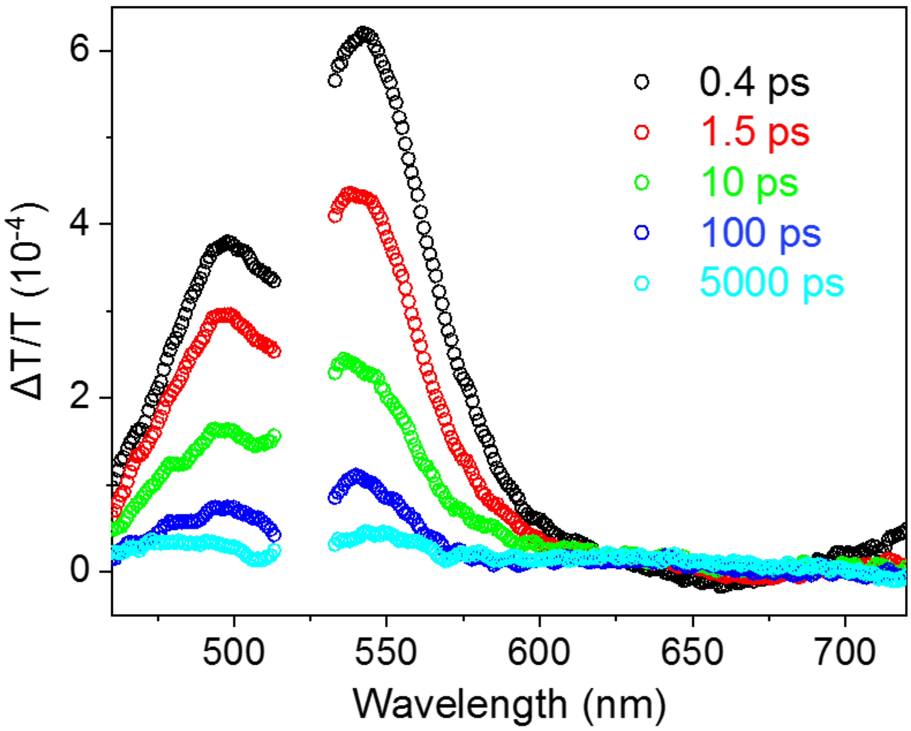

Figure S21. TA spectra of COP-BBT at different delay times after 525 nm excitation.

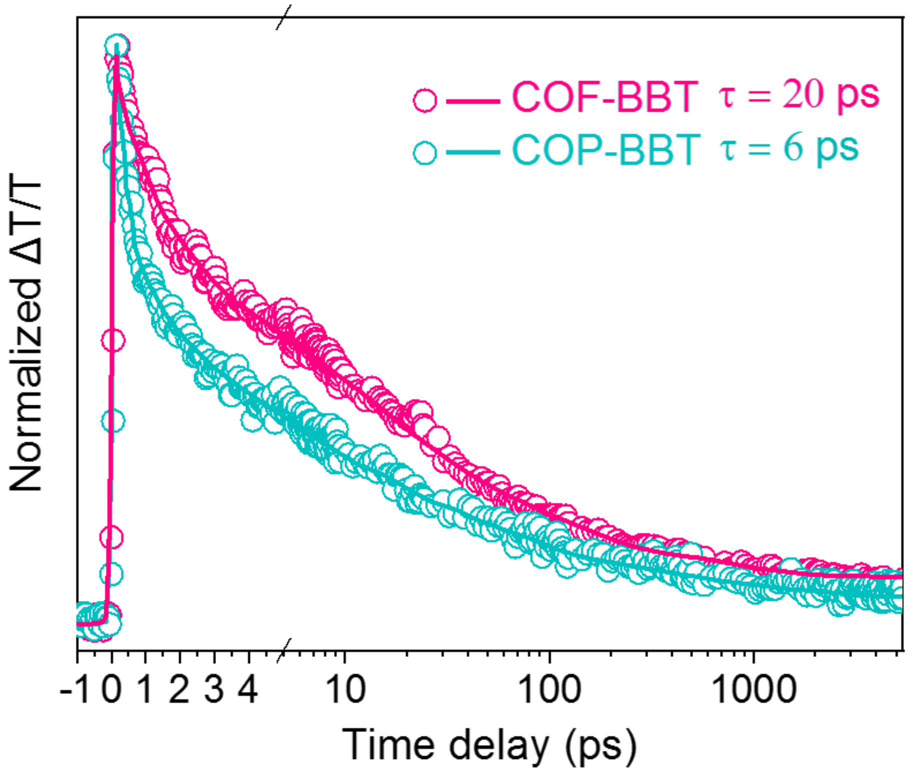

Figure S22. Normalized ESA signals (open circles) and fitting results of COF-BBT and COP-BBT probed at 560 nm and 625 nm, respectively. ESA signals are fitted with triexponential functions. The average excited state lifetime is estimated to be ~20 ps for COF-BBT and 6 ps for COP-BBT.

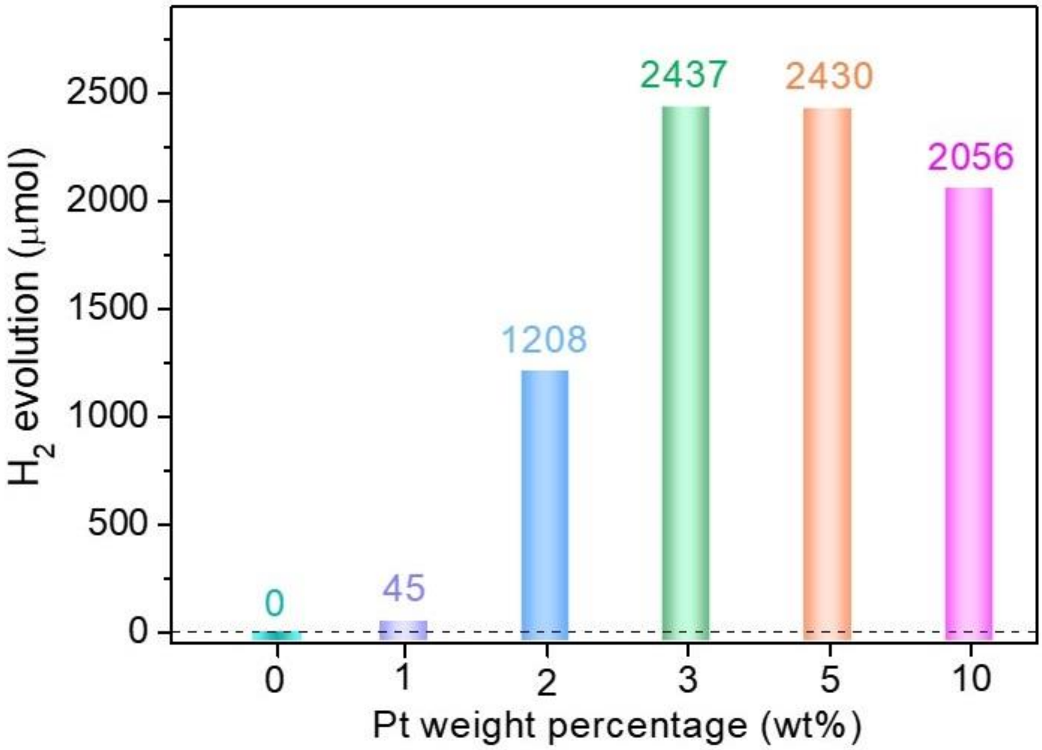

Figure S23. H_2 amount produced on COF-BBT loaded with different Pt weight percentages after the light irradiation for 5 h. (Reaction conditions: 10 mg catalyst, 50 mL 0.1 M ascorbic acid, 300 W Xe-lamp with a cutoff filter of $\lambda > 420$ nm).

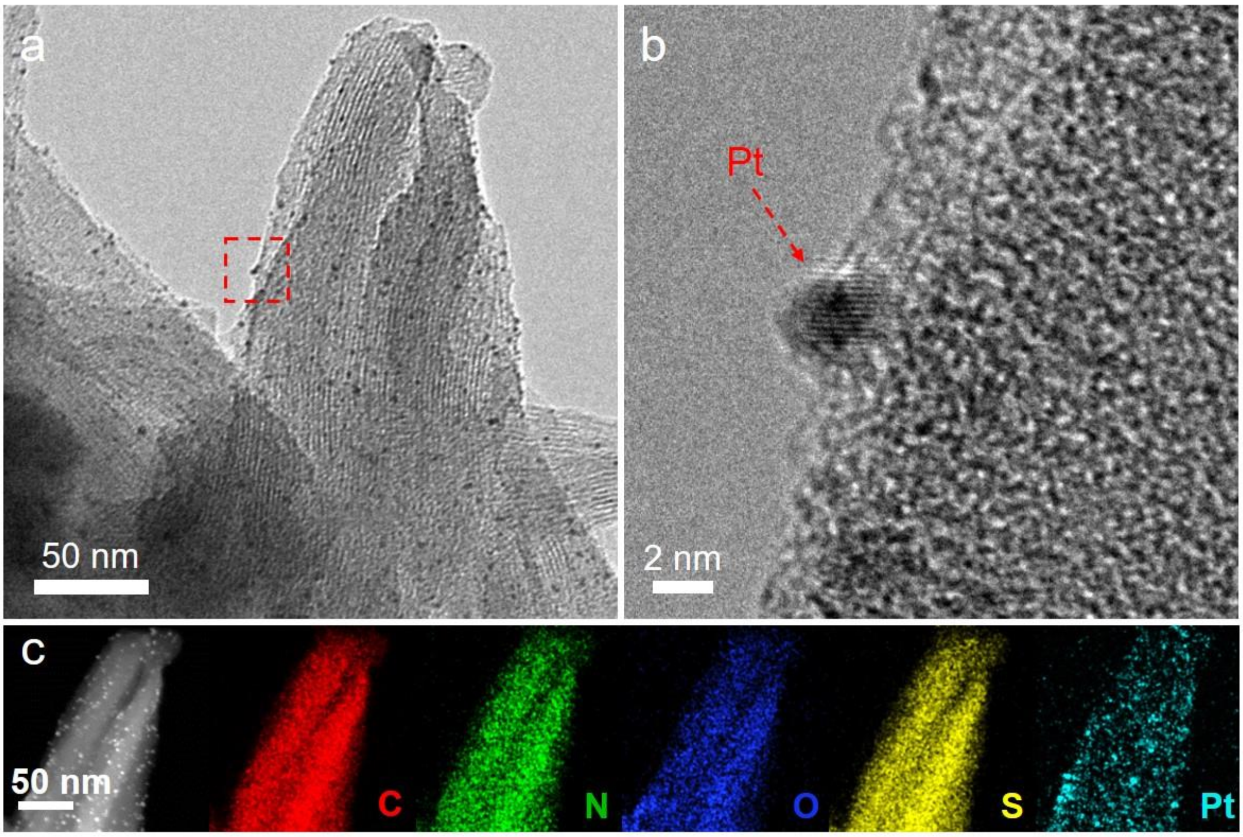

Figure S24. (a, b) High-resolution TEM images of COF-BBT with *in-situ* photodeposited Pt cocatalyst; (c) its EDS mapping.

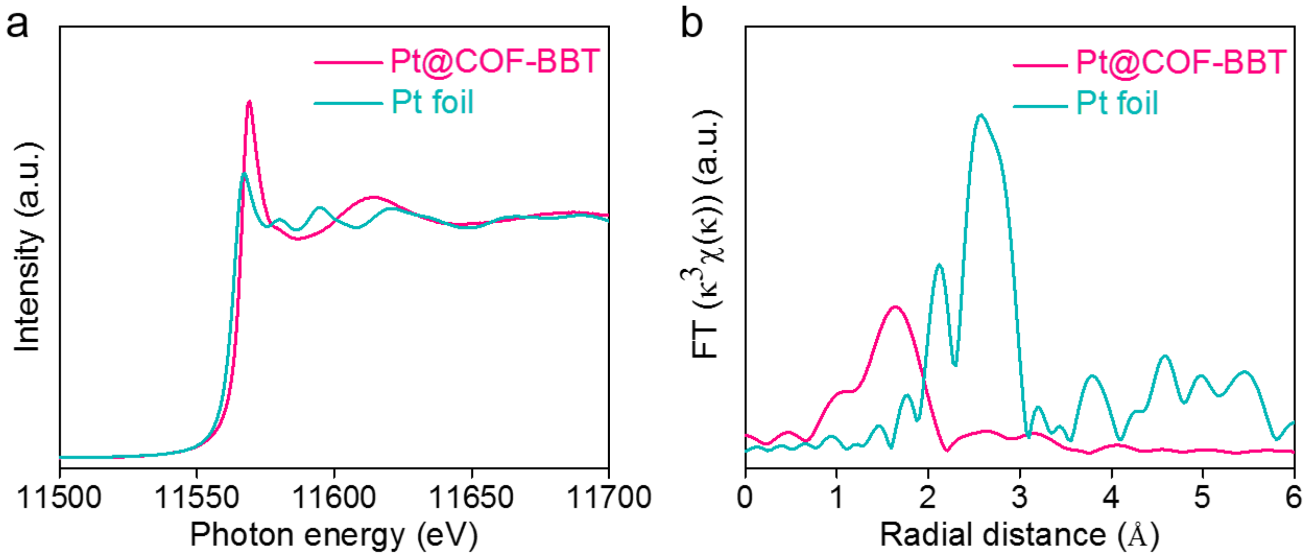

Figure S25. (a) Pt L_3-edge XANES spectra and (b) corresponding Fourier-transform EXAFS spectra of Pt-deposited COF-BBT and Pt foil. The Pt L_3-edge XANES spectrum of Pt-deposited COF-BBT is positively displaced compared to that of the Pt foil, suggesting that these Pt nanoparticles have a nonzero valence state. The corresponding EXAFS reveals a main signal at 2.58 Å likely assignable to the Pt-O bonding (*ACS Catal.* **2021**, *11*, 13266) although other possibilities could not be excluded. It evidences a possible interaction between Pt nanoparticles and oxygen atoms of the keto nodes. This is also interestingly consistent with our DFT calculations suggesting that LUMO electrons are enriched on the keto nodes and thereby can be readily transferred to Pt during photo-deposition.

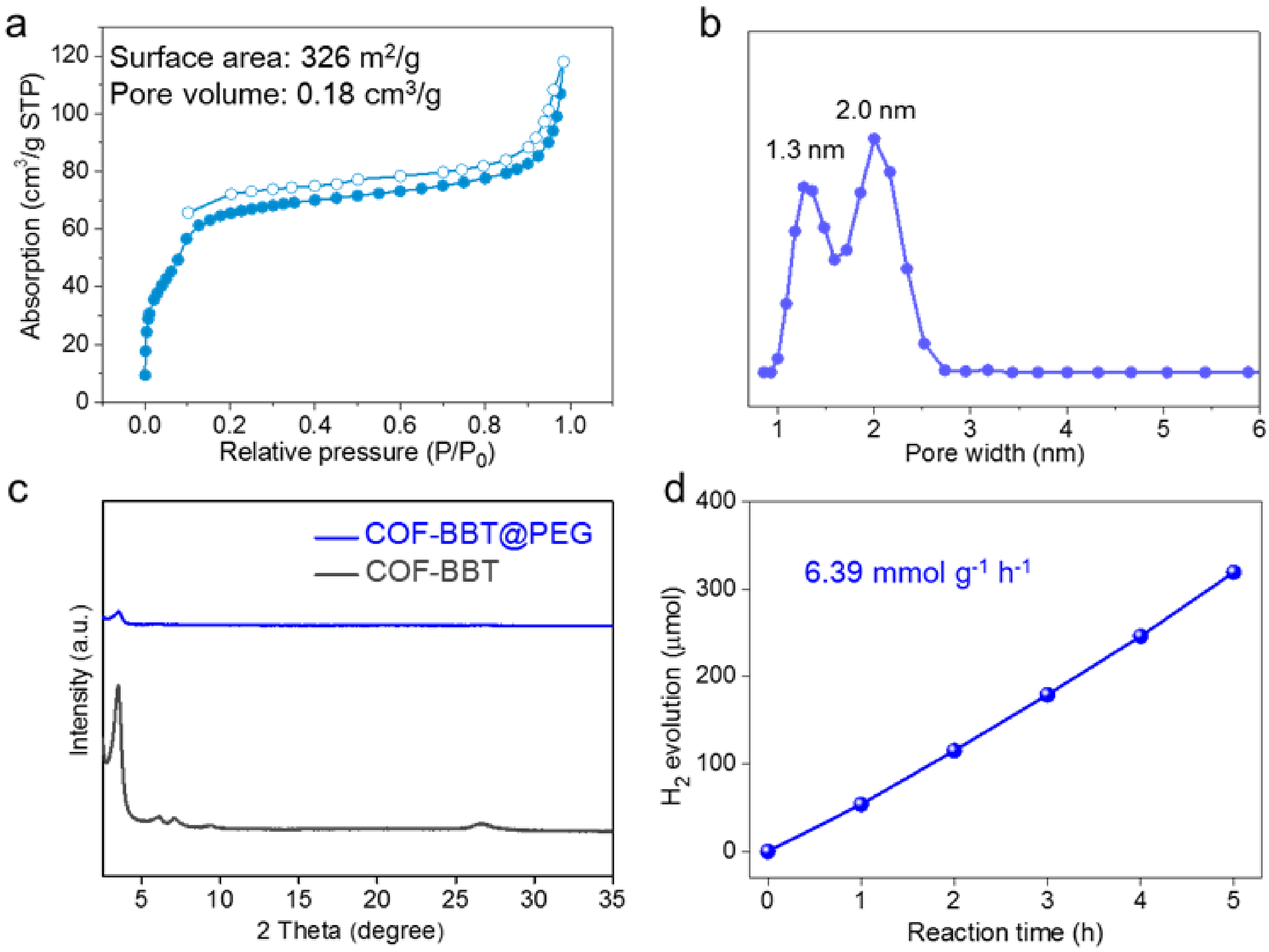

Figure S26. (a) N_2 isotherm of COF-BBT@PEG and (b) the corresponding pore size distribution curve; (c) XRD patterns of COF-BBT and COF-BBT@PEG; (d) photocatalytic H_2 production with COF-BBT@PEG;

Note: COF-BBT@PEG was prepared according to the literature (*Nat. Commun.* **2022,** 13, 2357). Simply, 40 mg of polyethylene glycol (PEG) was dissolved into 10 ml of tetrahydrofuran before 100 mg of COF-BBT was added. After stirred for 5 h under vacuum, the samples was separated by filtration, washed with tetrahydrofuran and dried under vacuum.

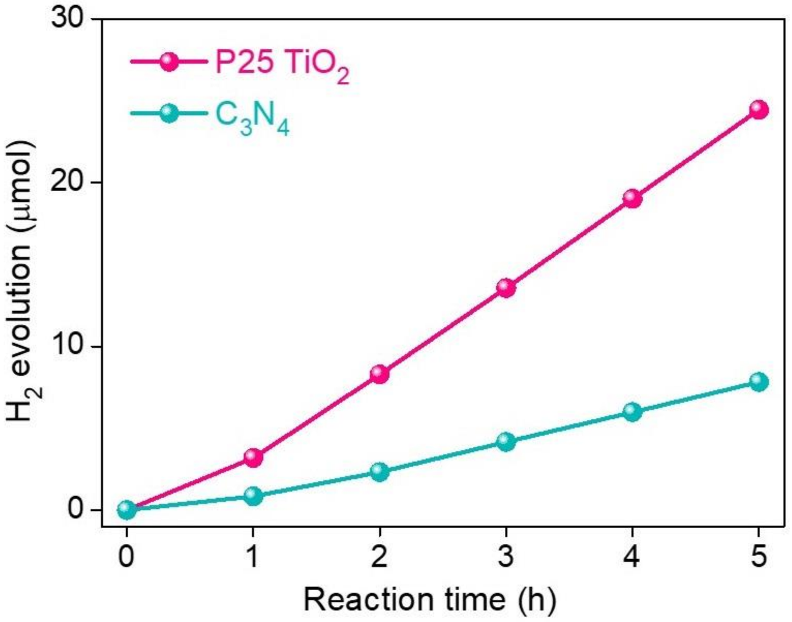

Figure S27. Photocatalytic H_2 production using commercial P25 TiO_2 and C_3N_4 under the identical condition. (Reaction conditions: 10 mg catalyst, 3 wt% Pt in 50 mL 0.1 M ascorbic acid, 300 W Xe-lamp with a cutoff filter of $\lambda > 420$ nm).

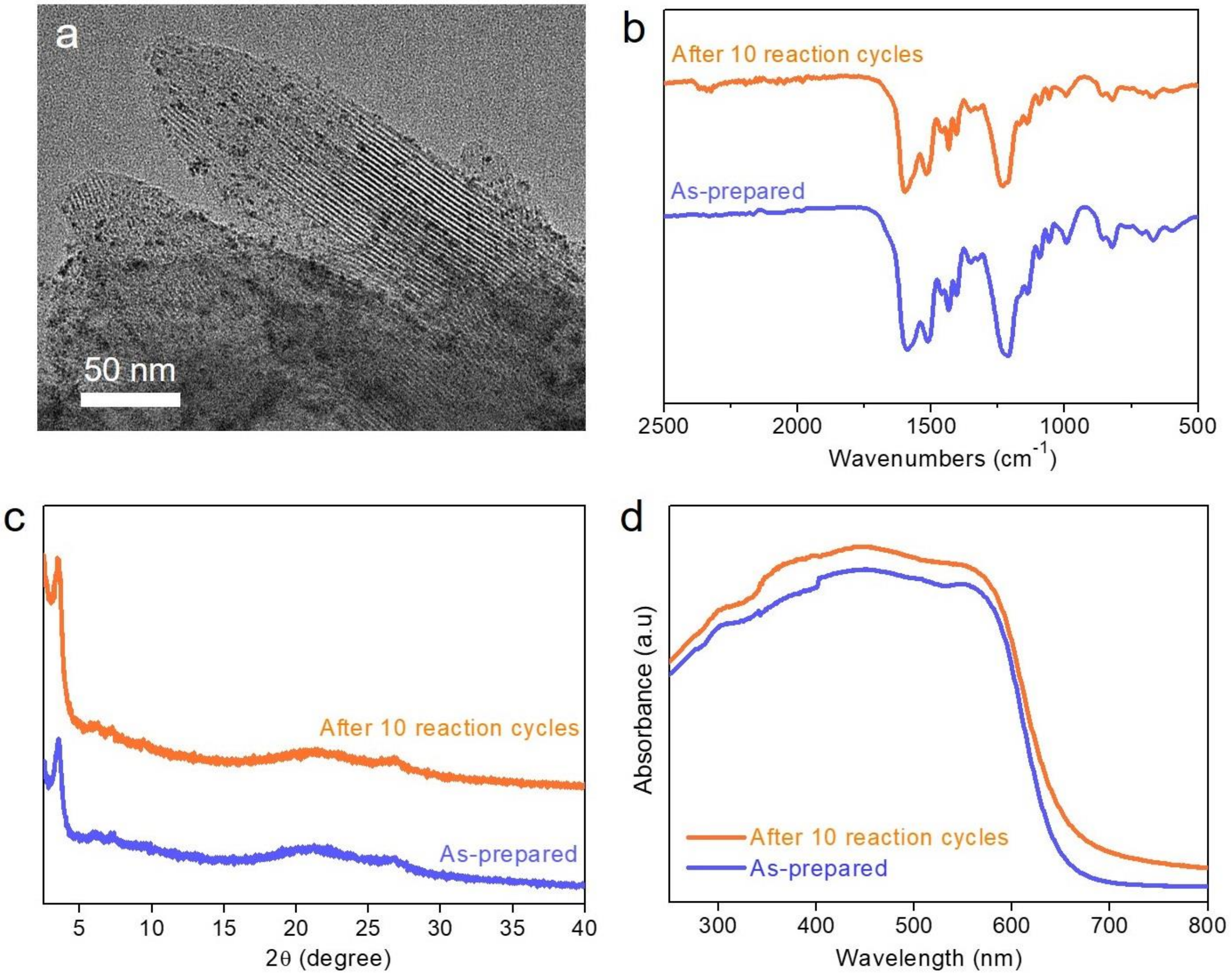

Figure S28. (a) TEM image of COF-BBT after 10 reaction cycles; (b) FT-IR spectrum, (c) XRD pattern and (d) UV-Vis diffuse spectrum of COF-BBT after 10 reaction cycles compared to those of the as-prepared sample.

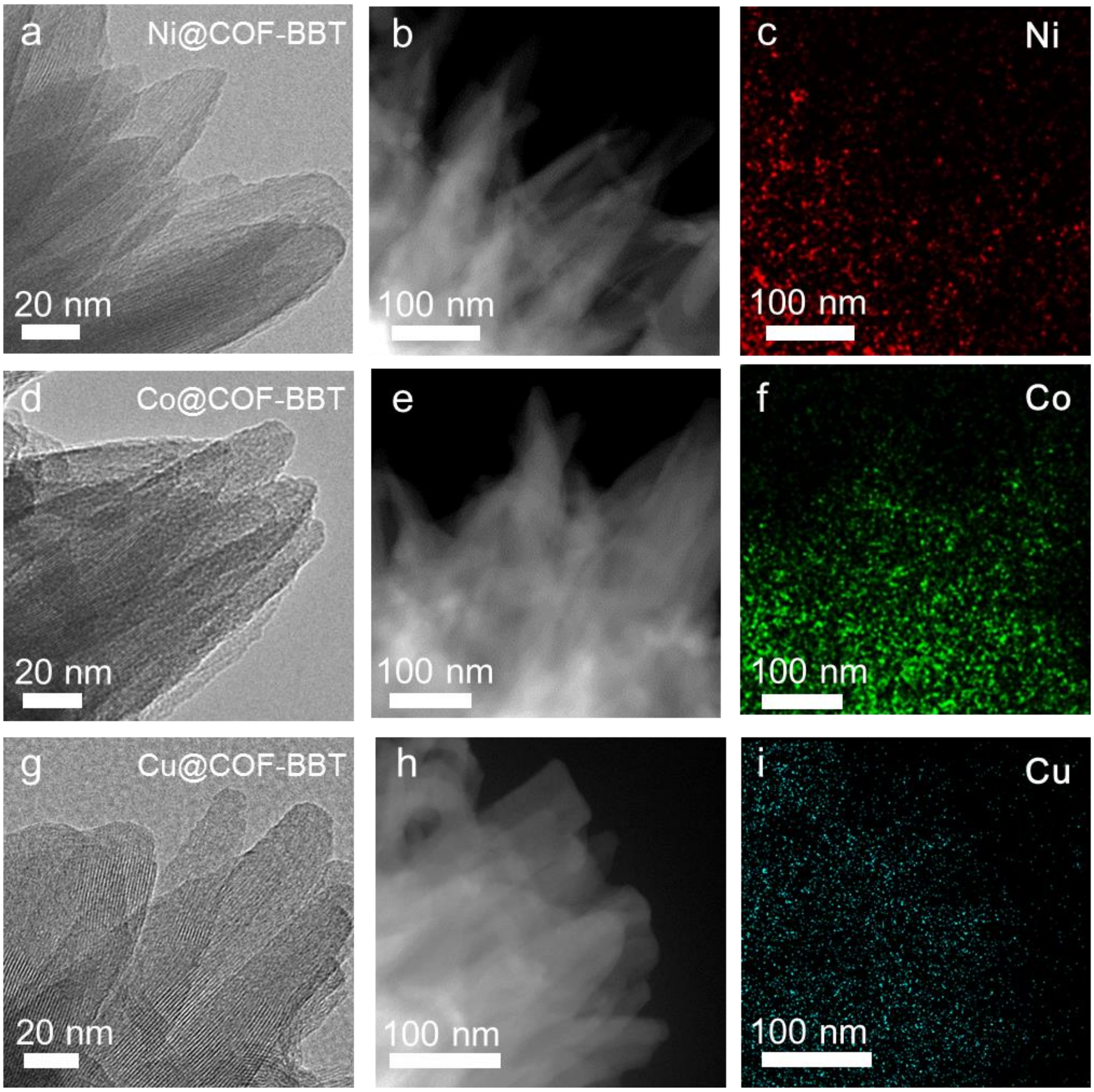

Figure S29. STEM images, HAADF-STEM images and EDS mapping of (a-c) Ni@COF-BBT, (d-f) Co@COF-BBT and (g-i) Cu@COF-BBT. To prepare samples, 15 mg of COF-BBT powder was dispsered in 20 mL of deionized H_2O and added with a calculated amount of $Co(NO_3)_2$, $Ni(NO_3)_2$ or $Cu(NO_3)_2$. After thorough mixing, the reaction solution was added with 0.5 mL of freshly prepared 0.2 M $NaBH_4$ solution, and stirred at 25℃ under N_2 for 2 h. The resultant M@COF-BBT was collected by centrifugation, washed with DI water and ethanol, and dried under vacuum at 60℃ for 8 h.

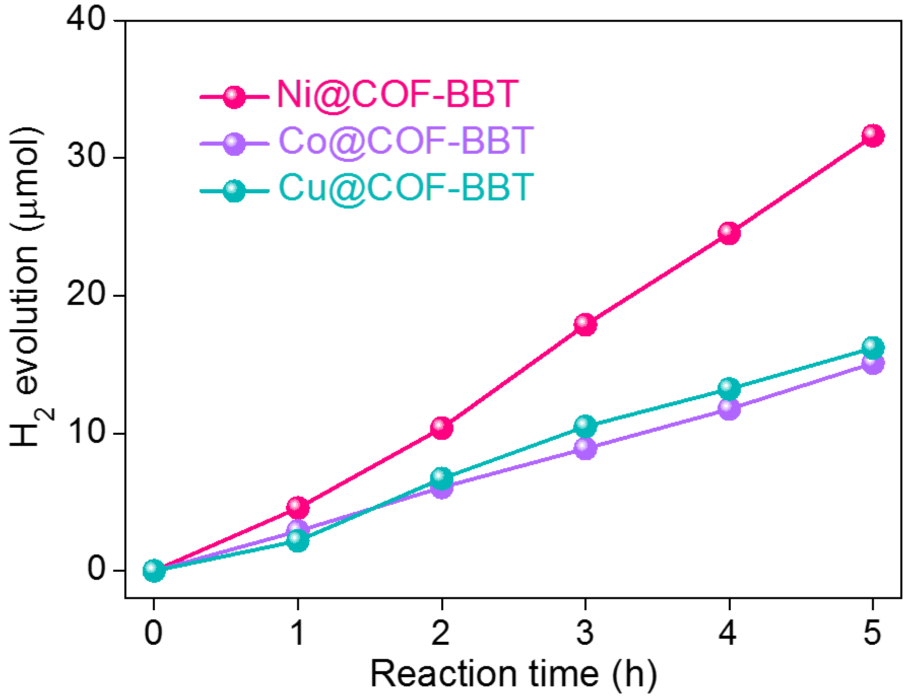

Figure S30. Photocatalytic H_2 evolution of M@COF-BBT (M = Ni, Co and Cu) under the standard condition (10 mg catalyst in 50 mL 0.1 M ascorbic acid, 300 W Xe-lamp with a cutoff filter of $\lambda > 420$ nm).

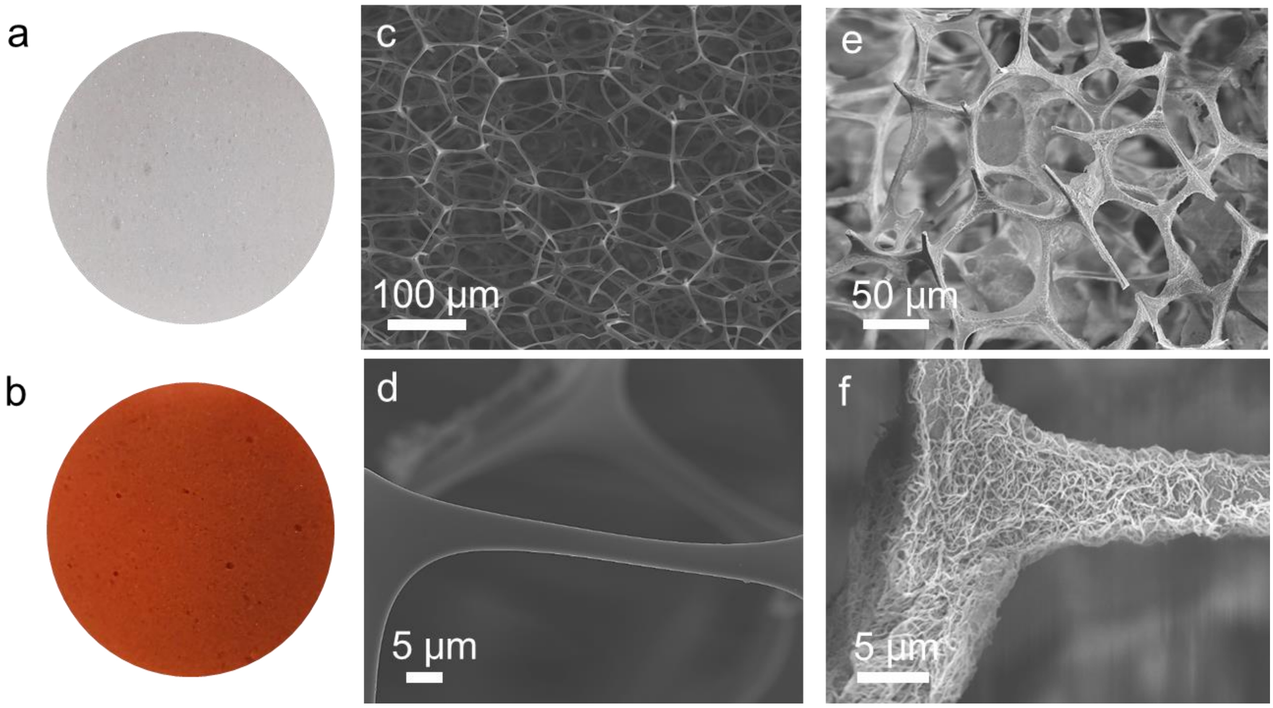

Figure S31. Photographs of (a) pristine melamine foam and (b) COF-BBT loaded melamine foam; (c, d) SEM images of pristine melamine foam at different magnifications; (e, f) SEM images of COF-BBT loaded melamine foam at different magnifications.

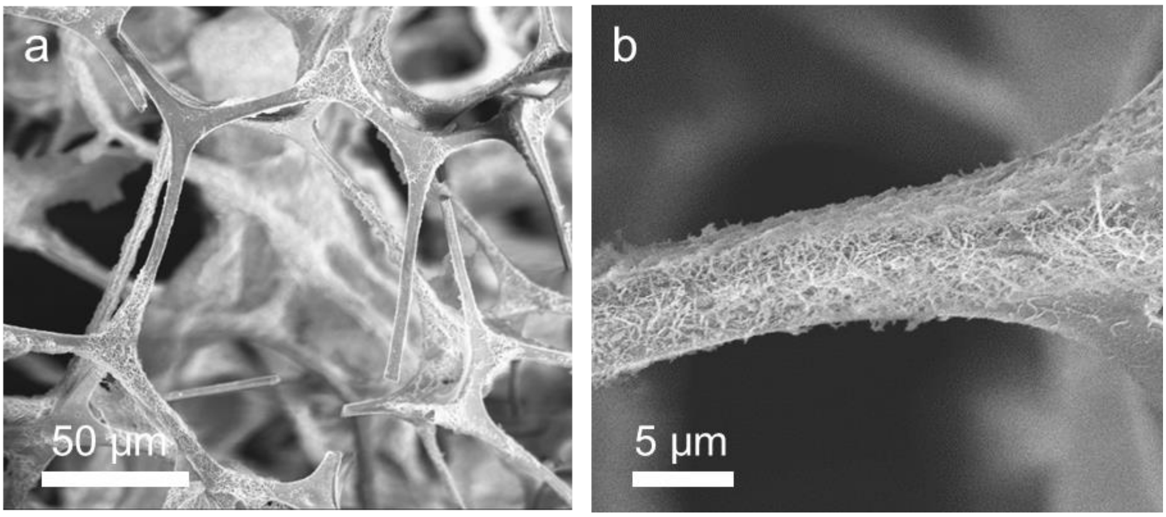

Figure S32. SEM images of catalyst-loaded melamine foam after 24 h photocatalytic reaction.

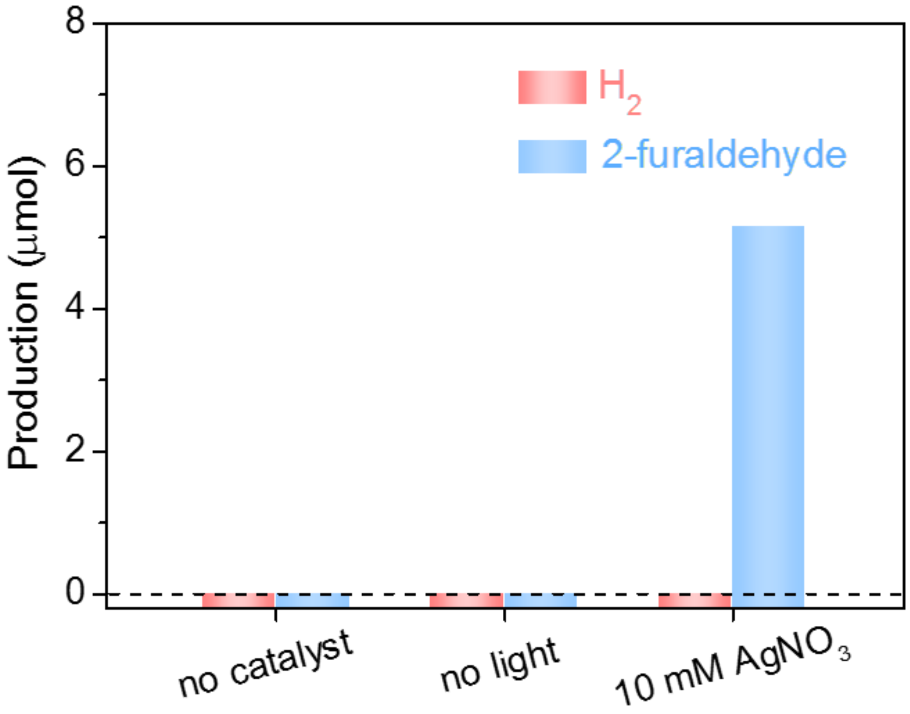

Figure 33. Production of H_2 and 2-furaldehyde after 5 h visible light irradiation under different reaction conditions as indicated.

Table S1. Comparison of COF-based materials for photocatalytic H_2 production.

Entry	PC	Light source	SED	Cocatalyst	Reaction rate (μmol $h^{-1}g^{-1}$)	EQE (%)	Ref
**1**	**COF-BBT**	**300 W Xe-lamp (λ>420 nm)**	**AA**	**Pt (3 wt%)**	**48800**	**6.8 (420 nm)**	**This work**
2	CYANO-CON	300 W Xe-lamp (λ>420 nm)	AA	Pt (1 wt%)	134200	82.6 (450 nm)	*Nat. Commun.* **2022**, 13, 2357.
3	PY-DHBD-COF	Xe-lamp (λ>420 nm)	AA	Pt (3 wt%)	71160 (in first 1 h) 45200 (in 5 h)	8.4 (420 nm)	*Nat. Commun.* **2022**, 13, 1355.
4	Tp-2C/BPy^{2+}-COF	300 W Xe-lamp (λ>420 nm)	AA	Pt (3 wt%)	34600	6.9 (420 nm)	*Angew. Chem. Int. Ed.* **2021**, 60, 9642-9649.
5	RC-COF-1	Xe-lamp (λ>420 nm)	AA	Pt (3 wt%)	27980	6.4 (420 nm)	*Nature.* **2022**, 604, 72-79.
6	TtaTfa	300 W Xe-lamp (λ>420 nm)	AA	Pt (8 wt%)	20700	1.43 (450 nm)	*Angew. Chem. Int. Ed.* **2021**, 60, 19797-19803..
7	TFPT-COF	300 W Xe-lamp (λ>420 nm)	TEOA	Pt (2.2 wt%)	1970	2.2	*Chem. Sci*., **2014**, *5*, 2789-2793.
8	CdS - COF	400 W Xe-lamp (λ>420 nm)	LA	Pt (0.5 wt%)	3678	4.2 (420 nm)	*Chem. Eur. J.* **2014**, *20*, 15961-1596
9	N_3-COF	(λ>420 nm)	TEOA	Pt (3 wt%)	1703	0.44 (450 nm)	*Nat. Commun.* **2015**, 6, 8508.
10	CTF-1-100W	(λ>420 nm)	TEOA+MeOH	Pt (2 wt%)	5500	6 (420 nm)	*Energy Environ. Sci.*, **2018**, *11*, 1617-1624.
11	CTF-HUST-C1	300 W Xe-lamp (λ>420 nm)	TEOA	Pt (3 wt%)	5100	--	*Angew. Chem. Int. Ed.* **2018**, 57, 11968–11972
12	TP-BDDA COF	300 W Xe-lamp (λ>395nm)	TEOA	Pt (3 wt%)	324±10	1.8 (520 nm)	*J. Am. Chem. Soc.* **2018**, 140, 1423−1427.
13	FS-COF	300 W Xe-lamp (λ>420 nm)	AA	Pt (3 wt%)	10100	3.2 (420 nm)	*Nature Chem*. **2018**, *10*, 1180-1189.
14	TpPa-1 COF/UiO-66	300 W Xe-lamp (λ>420 nm)	AS	Pt (3 wt%)	23410	--	*Angew. Chem. Int. Ed.* **2018**, *57*, 12106-12110
15	Pt-PVP-TP-COF	300 W Xe-lamp (λ>420 nm)	AA	Pt (6 wt%)	8420	0.4 (475 nm)	*Angew. Chem. Int. Ed.* **2019**, *58*, 18290-18294.
16	TpDTz	100 mW/cm^2 (AM 1.5)	TEOA	Ni(ME) (10 wt%)	941	--	*J. Am. Chem. Soc.* **2019**, *141*, 11082−11092
17	sp^2C-COF_{ERDN}	300 W Xe-lamp (λ>420 nm)	TEOA	Pt (3 wt%)	2120	0.48 (495 nm)	*Chem.* **2019**, *5*, 1632-1647.
18	*g*-$C_{33}N_3$-COF	300 W Xe-lamp (λ>420 nm)	TEOA	Pt (3 wt%)	292	1.06 (420 nm)	*J. Am. Chem. Soc.* **2019**, *141*, 14272−14279.
19	g-$C_{40}N_3$-COF	300 W Xe-lamp (λ>420 nm)	TEOA	Pt (3 wt%)	4120	4.84 (420 nm)	*Nat. Commun.* **2019**, *10*, 2467.
20	Py-ClTP-BT-COF	300 W Xe-lamp (λ>420 nm)	AA	Pt (5 wt%)	8875	8.45 (420 nm)	*Angew. Chem. Int. Ed.* **2020**, *59*, 16902-16909.

PC: photocatayst; SED: sacrificial electron donor; LA: lactic acid; AA: ascorbic acid; AS: ascorbic sodium; TEOA: triethanolamine.

Table S2. Sulfur concentration in solution before and after photocatalysis.

	Fresh solution	Recovered solution after photocatalysis
Sulfur concentration (ppm)	0.239 ±0.008	0.246 ±0.003

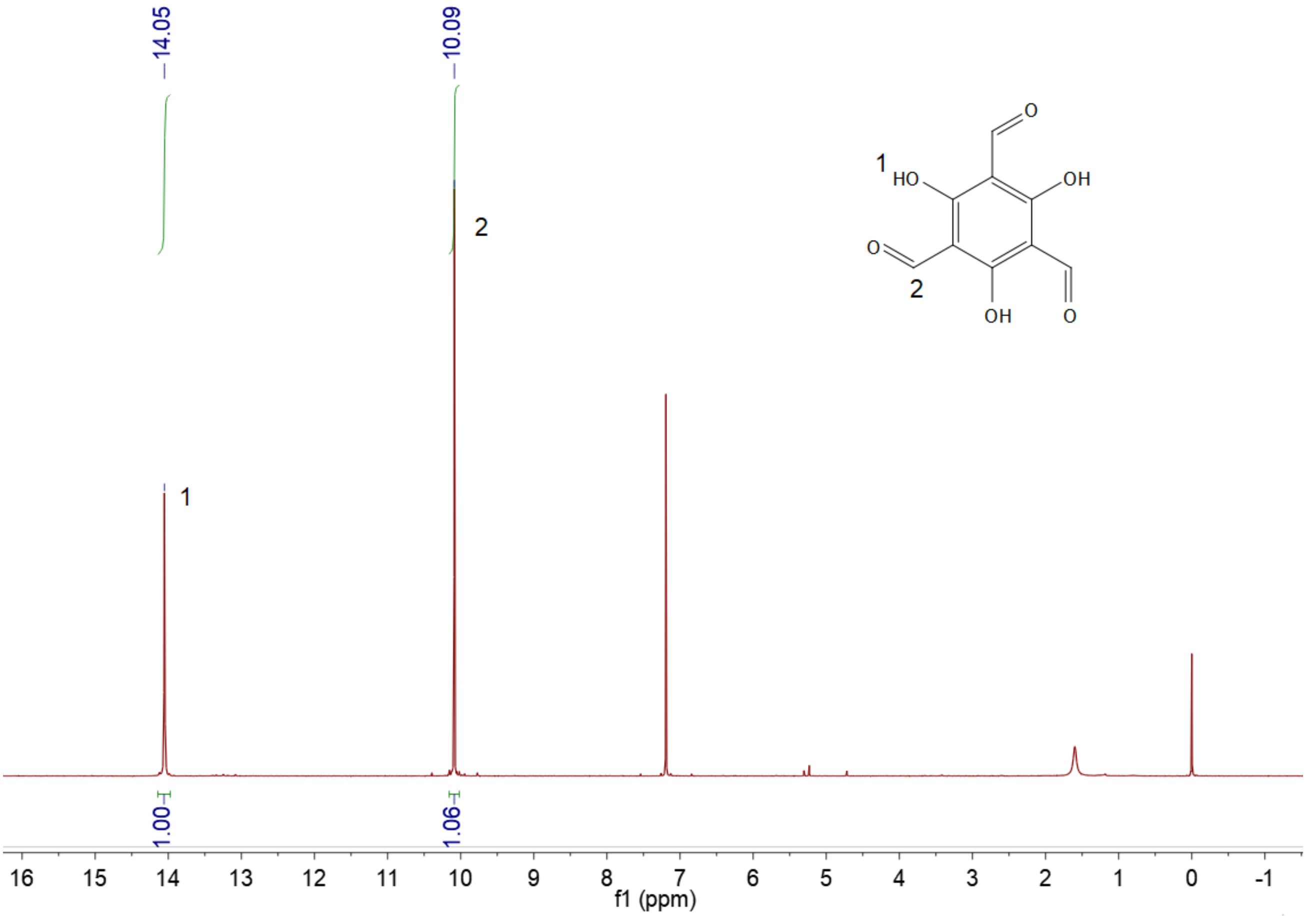
14.05
10.09
1
2
HO
OH
O
OH
1.00
1.06
16 15 14 13 12 11 10 9 8 7 6 5 4 3 2 1 0 -1
f1 (ppm)

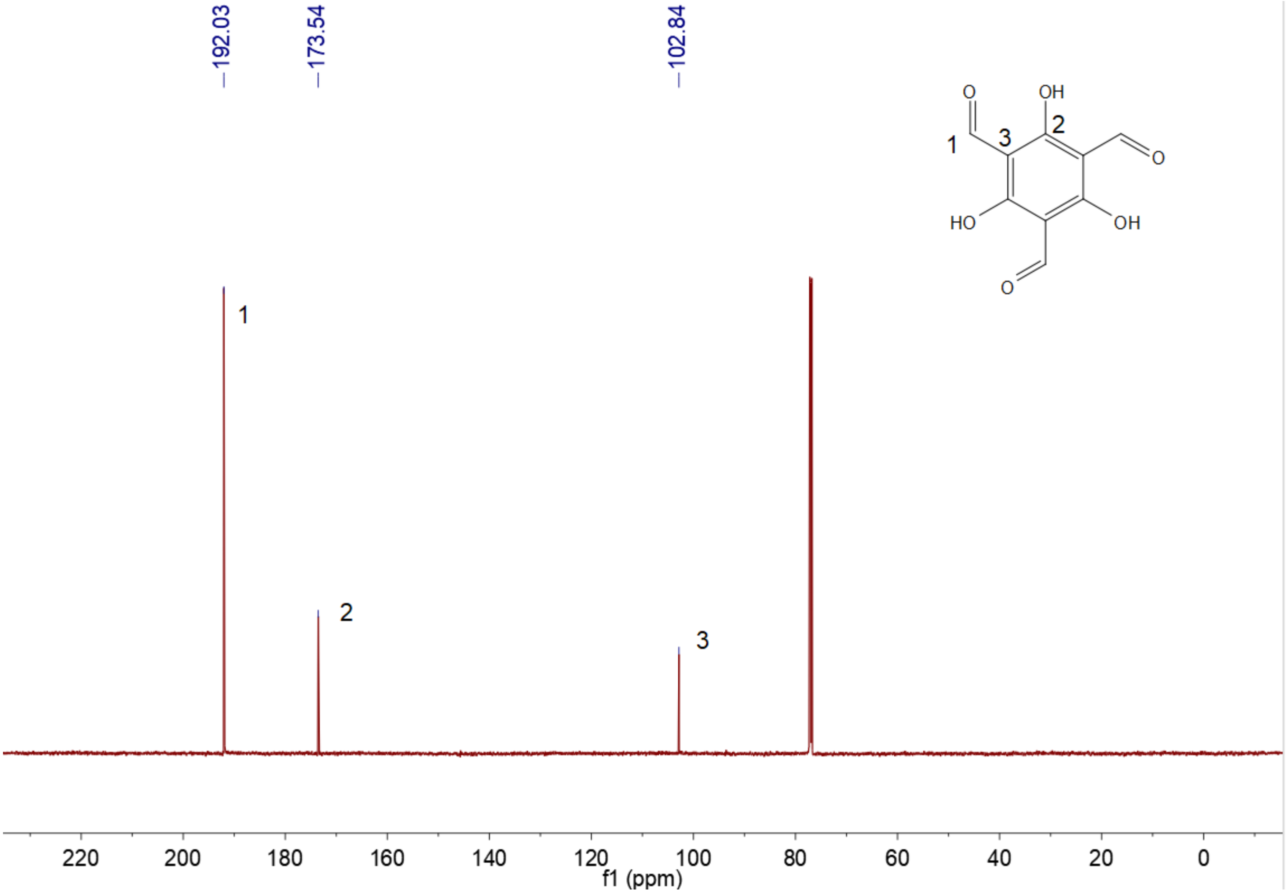
192.03
173.54
102.84
1
2
3
OH
HO
O
220 200 180 160 140 120 100 80 60 40 20 0
f1 (ppm)

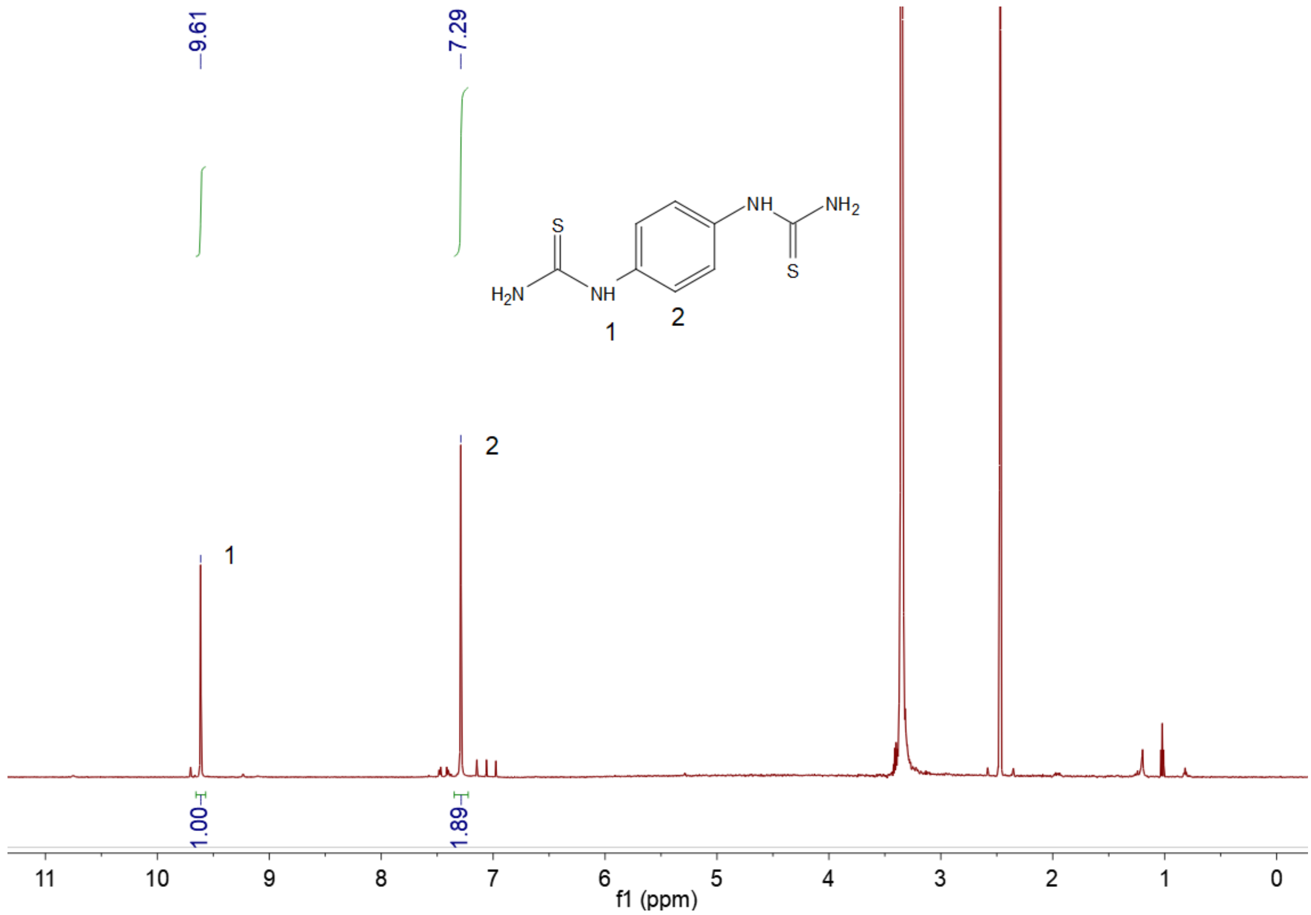

9.61
7.29
S
NH
NH2
H2N
NH
S
1
2
2
1
1.00
1.89
11
10
9
8
7
6
5
4
3
2
1
0
f1 (ppm)

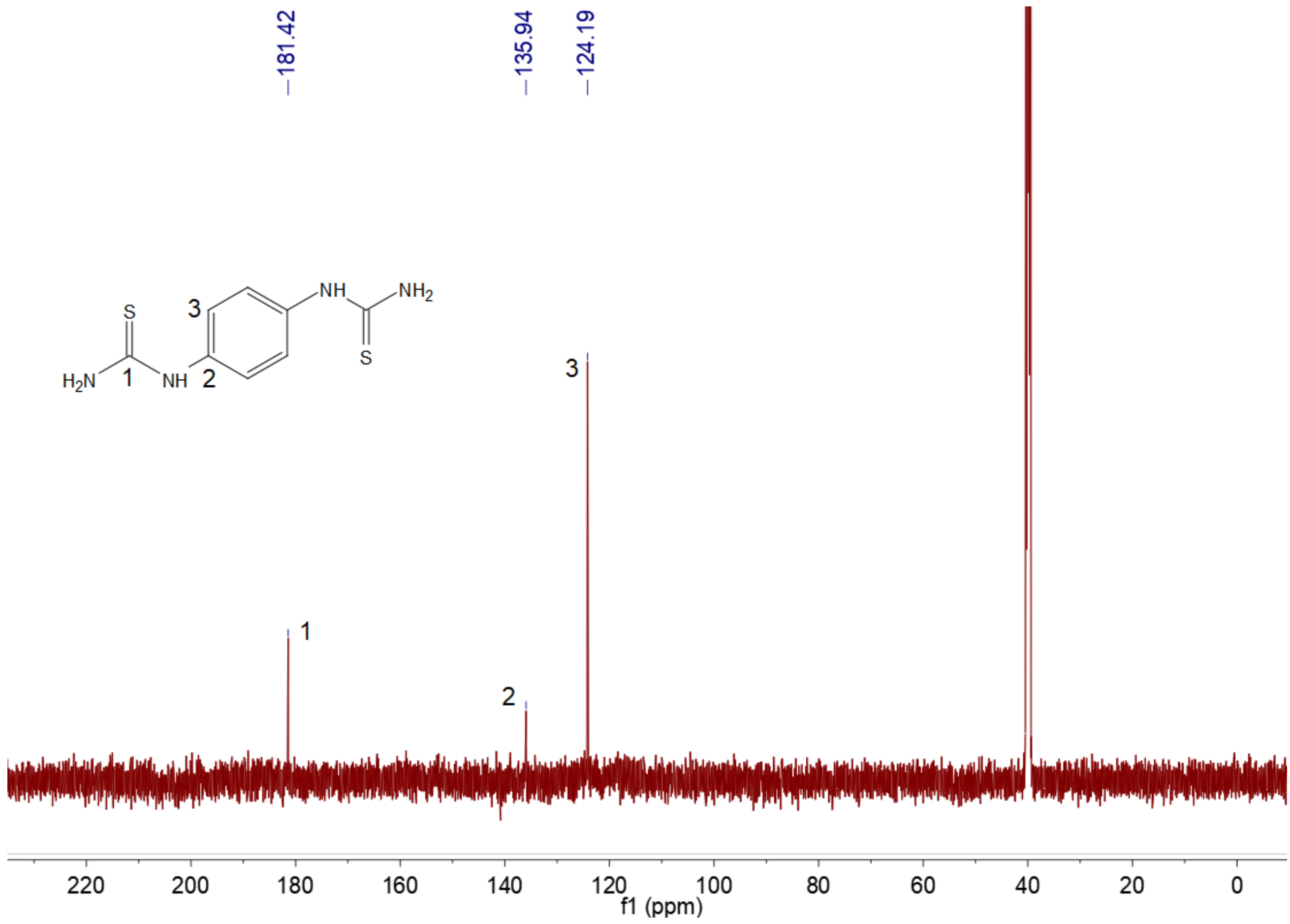

181.42
135.94
124.19
S
3
NH
NH2
H2N
1
NH
2
S
3
1
2
220
200
180
160
140
120
100
80
60
40
20
0
f1 (ppm)

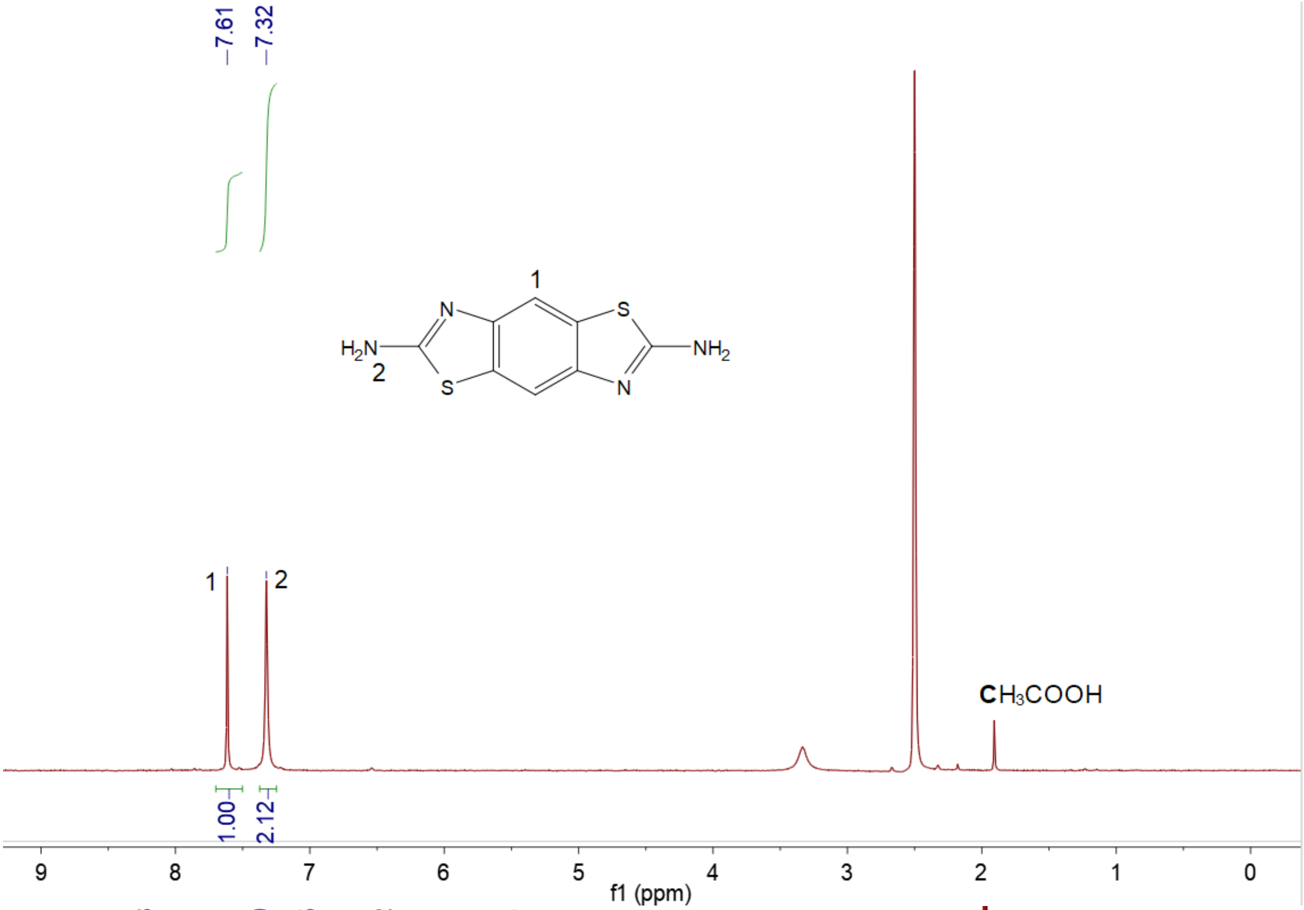

-7.61
-7.32
1
H2N
2
N
S
S
N
NH2
1
2
CH3COOH
1.00
2.12
9
8
7
6
5
4
3
2
1
0
f1 (ppm)

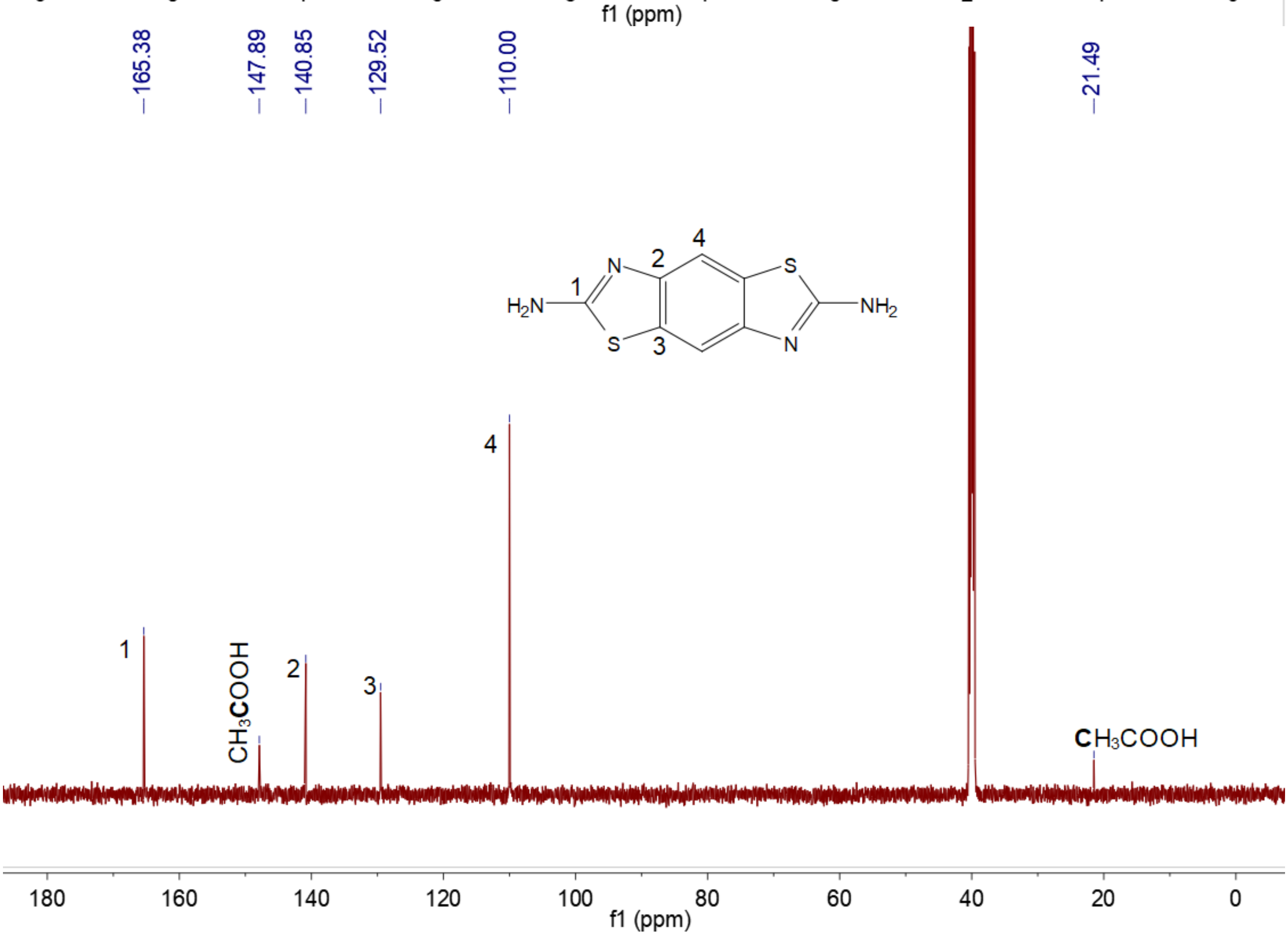

-165.38
-147.89
-140.85
-129.52
-110.00
-21.49
4
2
N
1
H2N
S
3
S
N
NH2
4
1
CH3COOH
2
3
CH3COOH
180
160
140
120
100
80
60
40
20
0
f1 (ppm)
